# Halide‐Free Synthesis of New Difluoro(oxalato)borate [DFOB]^−^‐Based Ionic Liquids and Organic Ionic Plastic Crystals

**DOI:** 10.1002/cphc.202200115

**Published:** 2022-05-17

**Authors:** Colin S. M. Kang, Oliver E. Hutt, Jennifer M. Pringle

**Affiliations:** ^1^ Institute for Frontier Materials Deakin University 221 Burwood Hwy Burwood Victoria Australia; ^2^ Boron Molecular 500 Princes Hwy Noble Park Victoria Australia

**Keywords:** difluoro(oxalato)borate, ionic liquids, organic ionic plastic crystals, synthesis, thermal and transport properties

## Abstract

The implementation of next‐generation batteries requires the development of safe, compatible electrolytes that are stable and do not cause safety problems. The difluoro(oxalato)borate ([DFOB]^−^) anion has been used as an electrolyte additive to aid with stability, but such an approach has most commonly been carried out using flammable solvent electrolytes. As an alternative approach, utilisation of the [DFOB]^−^ anion to make ionic liquids (ILs) or Organic Ionic Plastic Crystals (OIPCs) allows the advantageous properties of ILs or OIPCs, such as higher thermal stability and non‐volatility, combined with the benefits of the [DFOB]^−^ anion. Here, we report the synthesis of new [DFOB]^−^‐based ILs paired with triethylmethylphosphonium [P_1222_]^+^, and diethylisobutylmethylphosphonium [P_122i4_]^+^. We also report the first OIPCs containing the [DFOB]^−^ anion, formed by combination with the 1‐ethyl‐1‐methylpyrrolidinium [C_2_mpyr]^+^ cation, and the triethylmethylammonium [N_1222_]^+^ cation. The traditional synthetic route using halide starting materials has been successfully replaced by a halide‐free tosylate‐based synthetic route that is advantageous for a purer, halide free product. The synthesised [DFOB]^−^‐based salts exhibit good thermal stability, while the ILs display relatively high ionic conductivity. Thus, the new [DFOB]^−^‐based electrolytes show promise for further investigation as battery electrolytes both in liquid and solid‐state form.

## Graphical abstract

**The difluoro(oxalato)borate** [DFOB]^−^ anion is utilised for the synthesis of new ionic liquids and organic ionic plastic crystals, with the analysis of their thermal and transport properties. These neat electrolytes are synthesised via a halide‐free route to ensure highly pure materials.

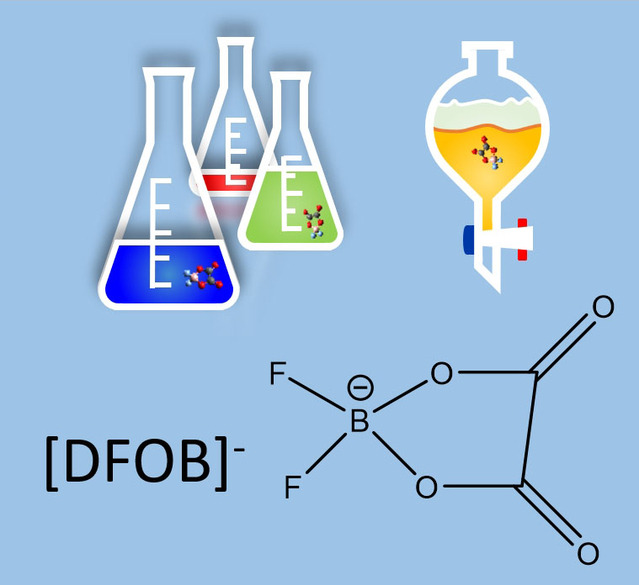

## Introduction

The progression of new battery technologies with higher energy densities, such as those with Lithium (Li) metal anodes, requires advanced electrolytes that do not suffer from decomposition nor present any safety hazards. Traditional organic electrolytes are known to break down in the presence of metallic Li, resulting in battery failure and fire risks. On the other hand, ionic liquids (ILs) and organic ionic plastic crystals (OIPCs) may play a pivotal role as emerging classes of advanced electrolytes due to their superior prospects for safety such as their high thermal/chemical stability, non‐flammability, and negligible vapour pressure.[^1^, ^2^] In addition, they are generally electrochemically stable over a wide potential range, particularly with the addition of lithium or sodium salts,^[3]^ which is beneficial for achieving full facilitation of high‐voltage cathode materials that would not usually reach their full potential with current organic electrolytes.[^4^, ^5^]

ILs are salts that consist of an organic cation and charge‐delocalised anion that melt below 100 °C, or below ambient temperature for room temperature ILs.^[6]^ ILs are often referred to as designer solvents due to the number of cation and anion combinations that allow unique tailoring of ions to achieve the desired properties.^[7]^ OIPCs are similar in that they are also composed entirely of ions, although they exist as solid‐state materials that exhibit long range order yet short‐range disorder. This disorder is believed to lead to vacancies and defects within the OIPC structure, allowing the transport of ions and enabling its inherent plasticity.^[8]^ These soft mechanical properties are also beneficial for ensuring good contact between the electrolyte‐electrode interface, particularly when volume expansion occurs.^[9]^

Furthermore, ILs and OIPCs can enable the transport of target ions, e. g. lithium or sodium, following the dissolution of such salts, which also results in orders of magnitude increases in ionic conductivity.[^9^, ^10^] Hence, the favourable properties of these electrolytes show potential for their use in next‐generation battery applications.

In current Li‐ion batteries, the lithium salt that is transported within the electrolyte utilises lithium hexafluorophosphate (LiPF_6_) (typically in ethylene carbonate/dimethyl carbonate (EC/DMC)). LiPF_6_ in this solvent system exhibits a balanced combination of properties such as a good ionic conductivity (and low dissociation energy), wide electrochemical window (>4 V), good solubility in alkyl carbonate solvents, chemical inertness to the battery cell components and an ability to form a protective stable passivation layer on the Al current collector to avoid corrosion.[^11^, ^12^] However, two major drawbacks are that: i) LiPF_6_ has low thermal stability such that degradation is accelerated at temperatures higher than 55 °C,^[12]^ which results in the formation of insoluble LiF and PF_5_ gas,^[13]^ and ii) LiPF_6_ is prone to hydrolysis when it is exposed to low concentrations of moisture and subsequently releases hydrogen fluoride (HF),^[14]^ which is highly toxic, and promotes further degradation of the battery.^[15]^ Despite these drawbacks, LiPF_6_ is currently used in commercial Li‐ion batteries. Hence, there is a strong need to find a new, safer lithium salt.

Alternative lithium salts, such as lithium bis(fluorosulfonyl)imide (LiFSI) and lithium bis(trifluoromethylsulfonyl)imide (LiTFSI), are widely used in the research field and display promising prospects as safer lithium salts. Both LiFSI and LiTFSI offer advantages such as higher thermal/chemical stability, moderate ionic conductivity, wider electrochemical window, high solvent solubility and greater resistance to hydrolysis when utilised in carbonate‐based solvents.[^16^, ^17^] However, LiTFSI is known to corrode the aluminium (Al) current collector due to its strong adsorption behaviour, forming Al(TFSI) species.[^18^, ^19^] Thus, LiTFSI requires the addition of an additive such as LiPF_6_ to achieve suitable Al passivation.^[17]^ In contrast, LiFSI can form a passivation layer on the Al current collector and prevent dissolution of Al, although the presence of trace impurities such as LiCl (∼50 ppm) will result in corrosion.[^16^, ^20^]

An alternative salt lithium difluoro(oxalato)borate (LiDFOB) forms a passivation film on the Al current collector, displaying superior inhibition against Al corrosion.[^21^, ^22^] Another important feature is that LiDFOB shows good Solid Electrolyte Interface (SEI) forming ability for Li‐ion batteries on graphite and Lithium Nickel Manganese Cobalt Oxide (NMC)‐based electrodes,[^21^, ^23^, ^24^] and in secondary Li‐metal batteries for Li deposition on a Cu/NMC electrode,[^25^, ^26^] resulting in low cell impedances and long cycling stability. Other advantageous properties of LiDFOB include its high salt solubility in carbonate‐based solvents, high thermal decomposition temperature, relatively high ionic conductivity, and that it does not form HF as a by‐product if hydrolysis occurs.[^21^, ^25^, ^27^] However, many studies have focussed on utilising LiDFOB dissolved in an organic electrolyte system, which still has drawbacks of flammability. As an alternative, utilising [DFOB]^−^ as the anion to form ILs or OIPCs could allow the benefits of both the aforementioned anion properties in addition to the non‐flammability and stability of ILs/OIPCs and open up new possibilities for electrolyte development. Thus far, only a limited number of ILs containing the [DFOB]^−^ anion have been synthesised, and its use has never been explored for OIPCs.

For the ILs that have been reported, the different combinations of cations that have been paired with the [DFOB]^−^ anion, and the resultant IL properties, are discussed below. Herzig *et al*.[^28^, ^29^] first reported the synthesis of a number of [DFOB]^−^‐based ILs such as those with the cations 1‐ethyl‐3‐methylimidazolium, [C_2_mim][DFOB], 1‐butyl‐3‐methylimidazolium, [C_4_mim][DFOB], and tetraethylammonium, [N_2222_][DFOB], through the reaction of oxalic acid, SiCl_4_ and the [BF_4_]^−^ salt (e. g. [C_2_mim][BF_4_], [N_2222_][BF_4_]). Using a similar method, Green and co‐workers paired the [DFOB]^−^ anion with two phosphonium cations, triisobutylmethylphosphonium [P_1i4i4i4_]^+^ and tributylethylphosphonium [P_2444_]^+^, to form two low‐melting solids and reported the latter to have a conductivity of 3×10^−3^ S cm^−1^ at 50 °C. Allen *et al*. synthesised 1‐butyl‐1‐methylpyrrolidinium [C_4_mpyr][DFOB], 1‐pentyl‐1‐methylpyrrolidinium [C_5_mpyr][DFOB] and 1‐hexyl‐1‐methylpyrrolidinium [C_6_mpyr][DFOB], through metathesis reactions with the respective bromide salt, and showed that none of these ILs caused corrosion on the Al current collector, even when doped with LiTFSI or LiFSI.^[30]^ Zhang and researchers extended this work and synthesised 1‐propyl‐1‐methylpyrrolidinium [C_3_mpyr][DFOB] through a similar synthetic procedure.^[31]^ They reported a melting point and decomposition temperature of 3 °C and 306 °C respectively, similar to that of [C_4_mpyr][DFOB]. Karuppasamy and co‐workers prepared [C_2_mim][DFOB], also by metathesis of the bromide salt.^[32]^ This work utilised the [DFOB]^−^‐based IL as a plasticiser in a gel polymer electrolyte system with LiDFOB and PVDF‐co‐HFP, and obtained an ionic conductivity of 3×10^−4^ S cm^−1^. Moreover, a relatively high transference number, which depicts the ratio of Li‐ion migration to the total ionic migration, of 0.37 was obtained.^[32]^ Liang *et al*. developed a [DFOB]^−^‐based electrolyte system consisting of 1‐methyl‐1‐propylpiperidinium [C_3_mpip][DFOB], LiTFSI and DMC that provided stability against Al corrosion and transition metal dissolution when using a Lithium Nickel Manganese Oxide and graphite electrode system.[^33^, ^34^]

In the aforementioned synthetic methods, undesired impurities may be present in the [DFOB]^−^‐based ILs which could have an effect on its physical properties. For example, Seddon *et al*. reported that the presence of halide impurities such as ∼0.5 mol kg^−1^ Cl^−^ in [C_4_mim][BF_4_] led to a 36 % increase in viscosity.^[35]^ Other properties such as ionic conductivity, electrochemical stability and thermal stability would likely be affected too.^[36]^ Hence, Allen *et al*. carried out ICP‐MS measurements and elemental analysis on their reported alkylpyrrolidinium [DFOB] ILs, and detected insignificant levels of Na^+^ and Br^−^ (i. e. <3 ppm).^[30]^ On the other hand, Herzig and co‐workers reported that their method, which uses SiCl_4_, resulted in traces of chloride and 0.5 % [BF_4_]^−^‐based impurities from the starting material.[^28^, ^37^] In other work, Schreiner *et al*. determined that 1–2 % of the [BF_4_]^−^ anion remained after synthesising [DFOB]^−^‐based ILs via the trimethylsilylated bi‐dentate reaction with [BF_4_]^−^‐based salts.[^23^, ^38^] Therefore, some synthetic procedures may be harder than others for achieving high purity, in which case it may be beneficial to consider alternative synthesis methods. It is worth noting here that while silver salts can lead to complete synthesis (due to the ease in removing silver halide by‐products),^[6]^ AgDFOB is not commercially available whilst also being expensive to make, which is particularly problematic with respect to scalability.

It is important to consider the possible disproportionation reaction where the [DFOB]^−^ anion can, under some conditions, disproportionate into [BF_4_]^−^ and bis(oxalato)borate [BOB]^−^. Studies carried out by Lucht *et al*. found that 1 M LiDFOB in EC/ethyl methyl carbonate (EMC) undergoes disproportionation and reaches equilibrium at approximately 82 % after it was left at 100 °C for 16 weeks.^[38]^ On the other hand, the same electrolyte system was found to be stable at ambient temperature, with no disproportionation observed. Amereller et al. found that LiDFOB, amongst other Li borate salts, undergoes hydrolysis in acetonitrile in the presence of water.^[39]^ In terms of a neat IL system, Allen and co‐workers observed that 96–97 % of the alkylpyrrolidinium [DFOB]^−^ ILs remained unchanged, whereas the other 3–4 % disproportionated into [BOB]^−^ and [BF_4_]^−^ anions.^[30]^ Hence, DFOB‐based salts have been shown to maintain their stability so long as moisture and high temperatures are avoided. Additionally, these disproportionation products may not be detrimental compared to such products including HF or PF_5_ gas, that are derived from LiPF_6_.

In this work, we report an alternative, halide‐free and [BF_4_]^−^‐free synthetic method for [DFOB]^−^‐based salts paired with small cations such as [P_1222_]^+^, [P_122i4_]^+^, [N_1222_]^+^ and [C_2_mpyr]^+^. These small cations have been utilised to raise the melting point to above room temperature and encourage OIPC formation. Prior work has shown these small cations to be beneficial for imparting advantageous properties such as high conductivity, high disorder and wide phase I ranges.[^40^, ^41^, ^42^] Here, we synthesise a range of hydrophilic salts through the use of tosylate [Ts]^−^‐based starting materials, foregoing the need for expensive silver salts. After determining their purity and confirming almost negligible anion disproportionation, the thermal behaviour was analysed and showed that [P_122i4_][DFOB] exists as an IL at room temperature, [P_1222_][DFOB] and [N_1222_][DFOB] are low‐melting OIPCs, and [C_2_mpyr][DFOB] is a high melting OIPC with a wide phase I range. The ionic conductivity of the studied [DFOB]^−^‐based salts display relatively high ionic conductivity, particularly once they have melted, partly due to their high ion dissociation.

## Results and Discussion

### Synthesis Route

**Figure 1:**
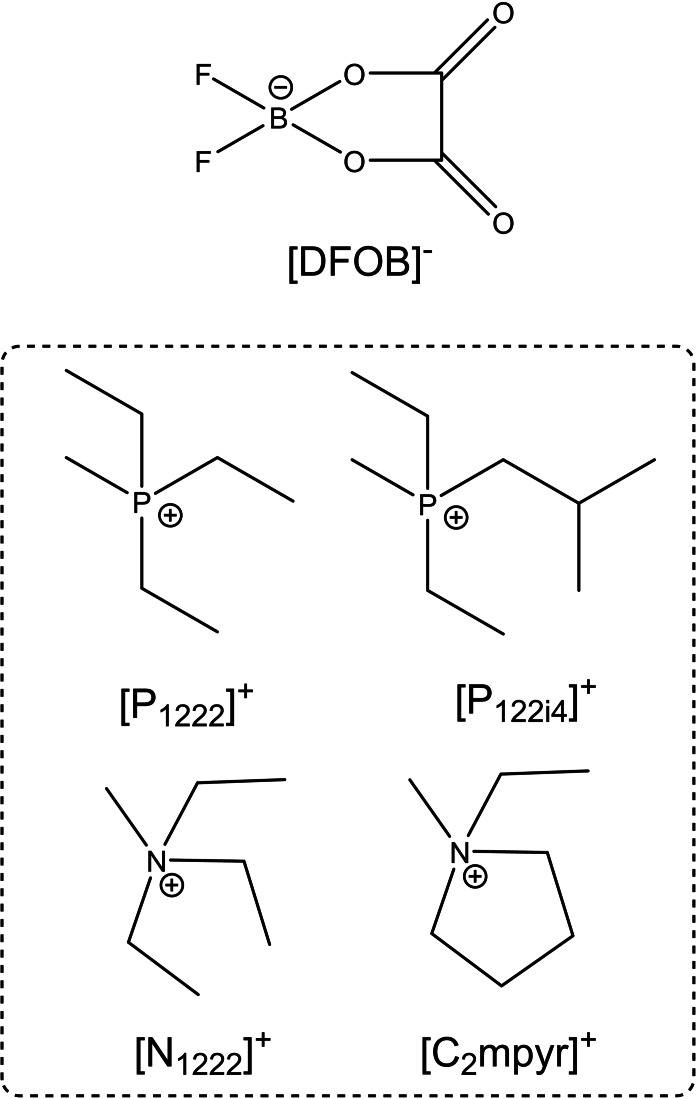
Structure of ions utilised in this study.

**Scheme 1:**
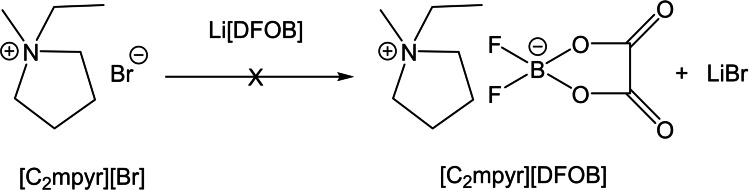
Initial Metathesis reaction for [C_2_mpyr][DFOB] via the halide route

Initially, the synthesis of DFOB‐based salts (Figure 1) was carried out in acetonitrile via the traditional halide metathesis route (Scheme 1).

However, persistent bromide‐based impurities remained in the final product that proved difficult to remove. One contributing factor is the partial solubility of LiBr in acetonitrile (8.8 g/100 g, 25 °C).^[43]^ Purification was not successful through biphasic extraction methods or by recrystallisation: due to the hydrophilic nature of [C_2_mpyr][DFOB], unwanted reaction species such as [C_2_mpyr][Br] and LiBr could not be separated from the desired product. In other attempts, the use of different solvents such as acetone, ethyl acetate, dichloromethane etc. did not result in an isolated product. An alternative synthetic route was explored using NaDFOB and acetone, since the by‐product NaBr is not highly soluble in acetone (1.2×10^−3^ mol L^−1^).^[44]^ However, although the desired product was formed, the bromide content was still high (>5000 ppm). Thus, despite the limited solubility of undesired bromide salt by‐products in common organic solvents (e. g. acetone, dichloromethane, ethyl acetate), they still exhibit some miscibility in the [DFOB]^−^ salt/organic solvent mixture. This issue was similarly experienced by Seddon and co‐workers, where metathesis reactions to synthesise imidazolidium‐based ILs via NaBF_4_/NaNO_3_ resulted in high residual chloride concentrations (>1 mol kg^−1^).^[35]^

A common strategy to overcome the problems associated with the solubility of alkali halide by‐products is to utilise Ag‐based salts,^[45]^ to precipitate Ag‐halide by‐products and drive the reaction forward. However, Ag[DFOB] is not commercially available, plus the use of expensive Ag salts is prohibitive to scale‐up. Therefore, we investigated an alternative route, via the tosylate salt, [C_2_mpyr][Ts]. In the current literature, [C_2_mpyr][Ts] is synthesised through a three‐step procedure:[^46^, ^47^]

- Quarternisation reaction of *N*‐methylpyrrolidine with bromoethane followed by recrystallisation
- Anion‐exchange from aqueous [C_2_mpyr][Br] to [C_2_mpyr][OH]
- Neutralisation reaction with tosylic acid

**Scheme 2:**
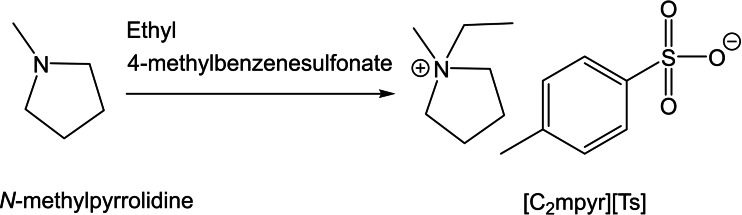
Synthesis of [C_2_mpyr][Ts] via quarternisation.

To consolidate the above reaction into one step, we can directly carry out the quarternisation reaction of *N*‐methylpyrrolidine with ethyl 4‐methylbenzene sulfonate (Scheme 2).

Although other quarternisation starting materials exist, such as methyl triflate, dimethyl sulfate, dimethyl carbonate,[^48^, ^49^] tosylate‐based starting materials were utilised due to the versatility of the nucleophilic substitution reaction from alcohols. For example, Kar *et al*. synthesised a range of unique alkylating agents via tosyl chloride in order to quarternise amines to form alkoxy‐based ammonium salts.[^45^, ^50^]

**Scheme 3:**
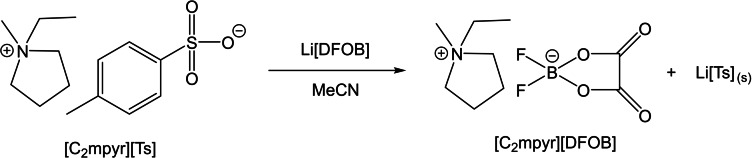
Metathesis reaction of [C_2_mpyr][DFOB] via the tosylate route.

After isolation of the tosylate salt with the desired cation (e. g. [C_2_mpyr][Ts]), the ion‐exchange metathesis reaction proceeds as shown in Scheme 3.

**Scheme 4:**
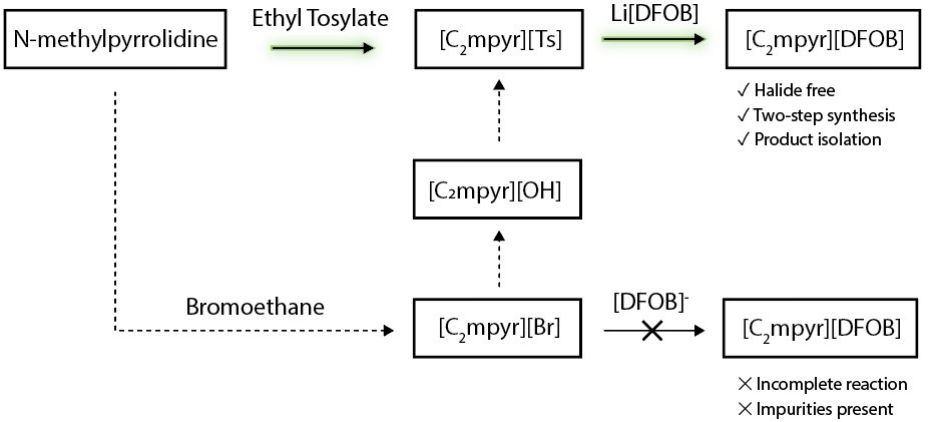
Summary of synthetic routes investigated for the preparation of [DFOB]^−^‐based salts.

The reaction is driven forward through the precipitation of the by‐product Li[Ts], which is insoluble in acetonitrile. Scheme 4 summarises the reaction routes investigated and highlights the benefits of the tosylate‐based route. The full reaction details are given in the experimental section.

### Long Term Stability

As discussed earlier, the presence of any undesired species can impact the properties of an electrolyte, particularly the long‐term stability of a battery electrolyte. Therefore, it is important to consider the possible disproportionation reaction of [DFOB]^−^ into [BOB]^−^ and [BF_4_]^−^ anions.[^30^, ^38^] That being said, these anions are not always pernicious and may not necessarily lead to battery failure, as would the formation of HF or PF_5_ gas. Nevertheless, we investigate the disproportionation reaction of our [DFOB]^−^‐based salts by measuring the ^19^F and ^11^B NMR as freshly prepared samples, and ‘aged’ samples (after 6 months storage under Ar at ambient temperature). The chemical shift of species are assigned as described in literature.[^23^, ^51^]

**Figure 2:**
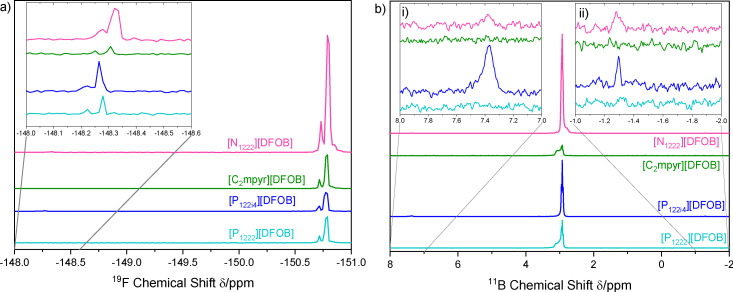
a) ^19^F and b) ^11^B NMR spectra of ‘aged’ [DFOB]^−^‐based salts stored at ambient temperature under Ar for 6 months. The inset in Figure 2a shows the ^19^F spectral range for [BF4]^−^ whereas the insets i) and ii) in Figure 2b show the ^11^B spectral range for [BOB]^−^ and [BF4]^−^ respectively.

The ‘aged’ ^19^F NMR spectra in Figure 2a shows where the [DFOB]^−^ anion exists at ∼−150.8 ppm, and the inset shows the spectral range at which the [BF_4_]^−^ anion would be present −148.3 ppm. Given the trace nature of the [BF_4_]^−^ peaks, it is obvious that a large proportion of the spectrum is composed of peaks from the [DFOB]^−^ anion, suggesting that the degree of disproportionation is very small. The ratios of each species are also tabulated in Table S1. In Figure 2b, the peaks in the ‘aged’ ^11^B NMR spectra at 7.4 ppm and −1.3 ppm are assigned to [BOB]^−^ and [BF_4_]^−^, respectively. Again, the [DFOB]^−^ peak at 3.0 ppm makes up the large majority of the spectrum, indicating that the extent of disproportionation reaction is very low at room temperature. Furthermore, no other ^19^F or ^11^B NMR peaks were observable in the spectra (e. g. BF(OH)_3_, BF_2_(OH)_2_, BF_3_OH) nor were any unknown ^1^H NMR peaks present. Thus, the purity of the ILs [N_1222_][DFOB], [C_2_mpyr][DFOB] and [P_1222_][DFOB] was identified to be greater than 99.4 %, whereas [P_122i4_][DFOB] was determined to exhibit a purity of 97.5 %.

Lastly, Figures S1‐S4 show that the spectra of ‘aged’ vs fresh samples are very similar, and that further disproportionation appears negligible during further storage (especially when handled in an argon atmosphere). Hence, through this synthetic route, there is very little disproportionation of the [DFOB]^−^ anion in the OIPCs/ILs.

## Thermal Behaviour

### Differential Scanning Calorimetry

The thermal properties of [DFOB]^−^‐based salts were examined to investigate their phase behaviour across a wide temperature range. The different solid phases observed are denoted as phase I, II, III etc., from the highest to lowest temperature phase, prior to the melt. While the size of the phase transition peaks can provide information on the disorder of a given OIPC, the temperature span of the solid phases will determine phase of the OIPC at a given temperature (e. g. if it is in most disordered phase I at the temperature of application). The melting point (T_m_), solid‐solid phase transitions (T_S‐S_) and the corresponding entropy change (ΔS) are shown in Table 1.

**Table 1:** Decomposition temperatures, phase transition temperatures and their associated entropy changes for [DFOB]^−^‐based salts.

	IV–III		III–II		II–I		I–Melt		T_d(onset)_ [°C]
	T_s‐s_ [°C] ±1 °C	ΔS_s‐s_ [J K^−1^ mol^−1^] ±10 %	T_s‐s_ [°C] ±1 °C	ΔS_s‐s_ [J K^−1^ mol^−1^] ±10 %	T_s‐s_ [°C] ±1 °C	ΔS_s‐s_ [J K^−1^ mol^−1^] ±10 %	T_m_ [°C] ±1 °C	ΔS_s‐s_ [J K^−1^ mol^−1^] ±10 %	
[P_1222_][DFOB]					−17	42	31	9	299
[P_122i4_][DFOB]					−13	52	10	23	299
[N_1222_][DFOB]			−6	12	5	25	50	15	298
[C_2_mpyr][DFOB]^[a]^	−43	19	4	16	28	52	198	10	298

[a] First cycle shown.

**Figure 3:**
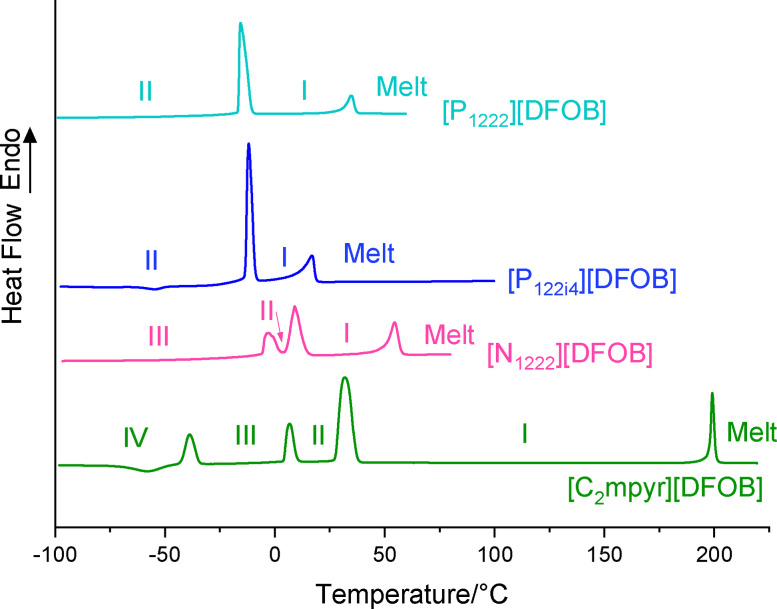
Differential Scanning Calorimetry traces for [DFOB]^−^‐based salts.

The DSC traces are shown in Figure 3, where all of the [DFOB]^−^‐based salts studied exhibit one or more phase transitions, which can be indicative of plastic crystal behaviour. However, with the relatively low melting points of [P_122i4_][DFOB] and [P_1222_][DFOB], 10 and 31 °C respectively, we would classify them to be more like ILs than OIPCs. The lower melting point of [P_122i4_][DFOB] compared to [P_1222_][DFOB] likely results from the larger size of alkyl chain decreasing ionic interactions, thus reducing its lattice energy. The other salts, [C_2_mpyr][DFOB] and [N_1222_][DFOB], melt at higher temperatures and are better defined as OIPCs. They also exhibit ΔS_f_ values below 20 J mol^−1^ K^−1^, and thus appropriately follow Timmerman's criteria for plastic crystal behaviour in molecular plastic crystals.^[52]^

The DSC traces confirm the formation of the first reported [DFOB]^−^‐based OIPCs, where [C_2_mpyr][DFOB] and [N_1222_][DFOB] exhibit multiple solid‐solid phase transitions and a low entropy of fusion. Interestingly, [C_2_mpyr][DFOB] displays a much higher melting point than [N_1222_][DFOB] (198 vs 50 °C respectively) with the sole difference being the cyclic structure of the pyrrolidinium cation vs the tetraalkylammonium cation. Hence, we define [N_1222_][DFOB] as an OIPC at room temperature, although we also measure its electrochemical window as an IL at 60 °C, discussed below. On the other hand, *N*‐alkyl‐*N*‐methylpyrrolidinium‐based [DFOB] salts, with a longer alkyl chain (where n=3, 4, 5, 6), reported by Zhang *et al*.^[31]^ and Allen *et al*.,^[30]^ display much lower melting temperatures and exist as ILs at room temperature. In terms of phase behaviour, [C_2_mpyr][DFOB] exhibits a wide phase I temperature range spanning over 150 °C whereas the range is less than 50 °C for [N_1222_][DFOB], again indicating an influence of the cyclic ring. The small exothermic events evident just below −50 °C for [C_2_mpyr][DFOB] and [P_122i4_][DFOB] are eliminated when cycled at a slower rate (2 °C/min, Figure S5) and thus likely represent a crystallization event that is not fully complete when a faster rate of cooling and heating is used.

[N_1222_][DFOB] exhibits slightly a higher melting temperature and displays an additional phase transition compared to [P_1222_][DFOB]. Thus, the nature of the central heteroatom has an impact on the thermal behaviour of the OIPCs. Blundell *et al*. and Carvalho *et al*., have suggested that cations with a phosphorus‐based cationic core tend to exhibit higher conformational flexibility and charge distribution compared to cations with a nitrogen‐based cationic core.[^53^, ^54^] Similarly, additional phase transitions were also observed in OIPCs [N_1222_][FSI] and [N_1222_][TFSI] in comparison to [P_1222_][FSI] and [P_1222_][TFSI].[^40^, ^41^]

Compared to [BF_4_]^−^‐based salts, those that contain the [DFOB]^−^ anion exhibit much lower melting points. For example, [C_2_mpyr][BF_4_]^[55]^ and [C_2_mpyr][DFOB] melt at 290 and 198 °C respectively, and [P_122i4_][BF_4_]^[40]^ and [P_122i4_][DFOB] melt at 136 and 10 °C respectively. This suggests that replacing two of the fluorine substituents on the boron anion with the larger oxalato functional group helps to decrease the electrostatic interactions with the ions. Although the melting points of [BOB]^−^‐based salts with these particular cations have not been reported in the literature, it is hypothesised that such salts would melt at temperatures between those of the [DFOB]^−^ and [BF_4_]^‐^‐based salts; prior work has shown that an increase in melting point occurs for [C_4_mpyr]^+^‐based salts in the order of the anions [DFOB]^−^<[BOB]^−^<[BF_4_]^−^ (−5, 55 and 143 °C respectively).[^30^, ^55^, ^56^] This trend was also observed in the tetraethylammonium salts, where [N_2222_][DFOB],^[28]^ [N_2222_][BOB]^[57]^ and [N_2222_][BF_4_]^[58]^ melt at 33, ∼130 and 365 °C, respectively. This suggests that the lower symmetry of the [DFOB]^−^ anion aids in decreasing the ionic interactions, thus results in lower melting points.

### Thermal Gravimetric Analysis

**Figure 4:**
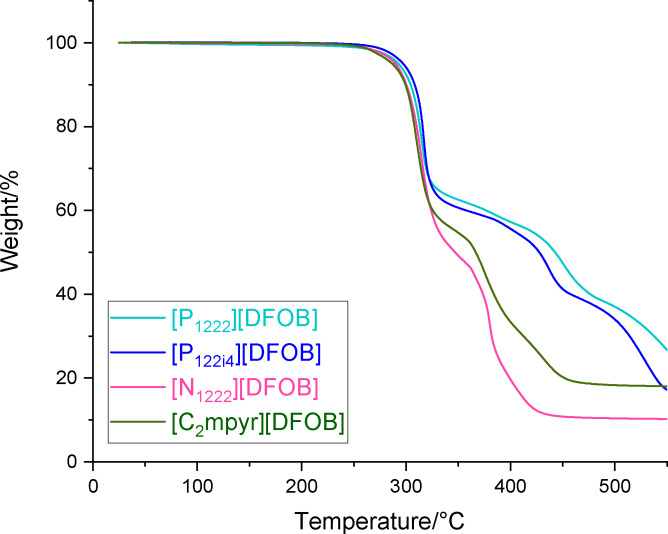
Thermal gravimetric analysis of [DFOB]^−^‐based salts.

The thermal decomposition temperature is often used to initially assess the upper temperature limit of an electrolyte for use in applications. In this work, the thermal stability was analysed under dynamic conditions by thermogravimetric analysis (TGA) under N_2_ using a heating rate of 10 °C min^−1^. The onset of decomposition (Table 1) was defined using the step‐tangent method, which provides an approximate assessment of decomposition temperature. In future work, to ascertain long term thermal stability, isothermal testing of the most promising materials would be used, as dynamic conditions can result in an overestimate of stability.[^59^, ^60^] As shown in Figure 4, all the [DFOB]^−^‐based salts studied undergo a multi‐step thermal decomposition, with similar onset temperatures at 299 °C for [P_1222_][DFOB] and [P_122i4_][DFOB], and 298 °C for [N_1222_][DFOB] and [C_2_mpyr][DFOB]. This likely indicates that the breakdown of the [DFOB]^−^ anion occurs prior to the cation and that the type of cation does not largely influence this breakdown temperature. Other *N*‐alkyl‐*N*‐methylpyrrolidinium‐based [DFOB] salts, reported by Zhang *et al*.^[31]^ and Allen *et al*.,^[30]^ were observed to exhibit similar thermal decomposition temperatures, indicating that the length of alkyl chain on the cation does not affect decomposition temperature.

For comparison, while [N_1222_][DFOB] thermally decomposes at ∼300 °C, Nanbu *et al*. reported the thermal decomposition temperature of [N_1222_][BOB] and [N_1222_][BF_4_] to be ∼250 °C and ∼325 °C respectively. This suggests that the fluorinated substituents help with thermal stability such that [BF_4_]^−^ is more stable than [DFOB]^−^ followed by [BOB]^−^.^[57]^ This same trend was also observed by Schmitz *et al*. where imidazolium‐based salts paired with [BF_4_]^−^ and [BOB]^−^ exhibited an onset of thermal decomposition at ∼330 °C and ∼250 °C respectively.^[61]^ Nonetheless, the TGA results show that these [DFOB]^−^ based salts are sufficiently thermally stable to allow their use as electrolytes in battery applications.

## Transport Properties

### Ionic Conductivity

**Figure 5:**
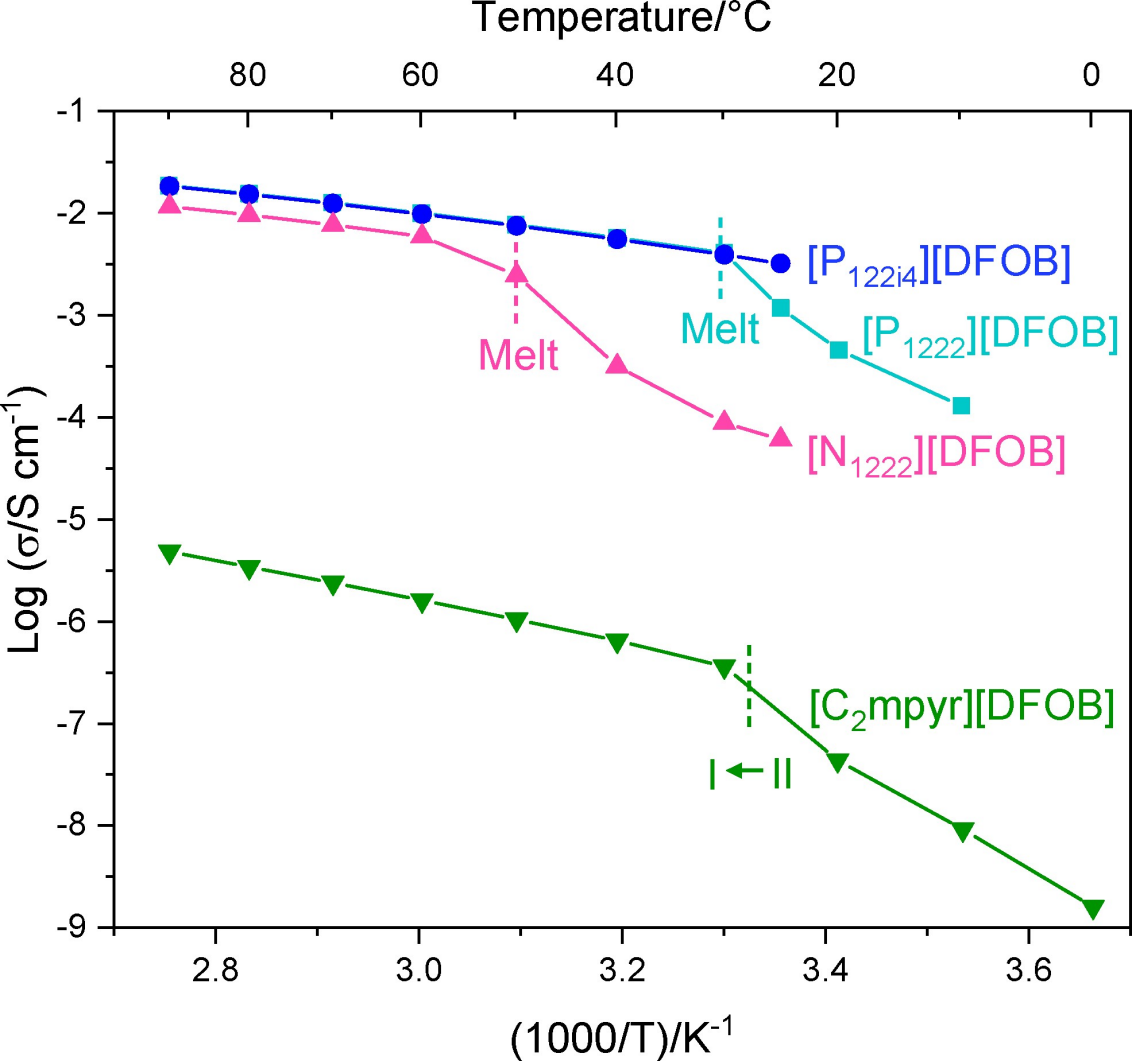
Plot of ionic conductivity for [DFOB]^−^‐based salts in this study.

The ionic conductivity is an important criterion for electrochemical applications and different cations of the IL/OIPC can have a strong influence on this parameter. Figure 5 shows the ionic conductivities of the [DFOB]^−^‐based electrolytes, which greatly differ depending on if the electrolyte is an IL or OIPC at a given temperature. The solid‐state OIPC [C_2_mpyr][DFOB] increases in ionic conductivity between 10–30 °C, across its solid‐solid phase II−I transition. At 30 °C, the ionic conductivity of [C_2_mpyr][DFOB] is 3.6×10^−7^ S cm^−1^; in comparison, Iranipour *et al*. reported the ionic conductivity of [C_2_mpyr][BF_4_] in phase II to be 8.0×10^−7^ S cm^−1^.^[62]^ Although the ionic conductivity of these neat salts are relatively low, it is worth noting that doping [C_2_mpyr][BF_4_] with LiBF_4_ led to an increase in conductivity such that this electrolyte system now has demonstrated applicability in lithium metal batteries.[^62^, ^63^] Hence, the new [DFOB]^−^‐based materials are still promising for the development of new electrolytes, particularly for high termperature applications.

In terms of the cation, a change in its heteroatom also results in a difference in ionic conductivity of the ILs, where the phosphonium IL [P_1222_][DFOB] shows ∼70 % higher ionic conductivity compared to the [N_1222_][DFOB] above the melt (1.0×10^−2^ S cm^−1^ vs 5.9×10^−3^ S cm^−1^ respectively, at 60 °C). In terms of the effect of the cation alkyl chains, an isobutyl substituent on the phosphonium cation instead of an ethyl group has negligible effect on the ionic conductivity: these are 4.1×10^−3^ S cm^−1^ and 3.9×10^−3^ S cm^−1^ for [P_1222_][DFOB] and [P_122i4_][DFOB] respectively, at 30 °C. Interestingly, the viscosity of [P_122i4_][DFOB] is higher than that of [P_1222_][DFOB] (86 vs 55 mPa S at 30 °C, respectively, Figure S6). This deviation from a linear relationship between ionic conductivity and viscosity is attributed to differences in ion dissociation, as illustrated by the Walden plot and discussed below.

For comparison with prior reports in the literature: the pyrrolidinium‐based ILs [C_3_mpyr][DFOB], [C_4_mpyr][DFOB] and [C_2o1_mpyr][DFOB] exhibit ionic conductivities of 2.2×10^−3^ S cm^−1^ (at 20 °C),^[31]^ 2.4×10^−3^ S cm^−1^ (at 25 °C)^[29]^ and 2.7×10^−3^ S cm^−1^ (at 25 °C),^[29]^ respectively. A few imidazolium‐based ILs, such as [C_2_mim][DFOB] and [C_4_mim][DFOB], display slightly higher ionic conductivities of 8.4×10^−3^ S cm^−1^ (at 30 °C) and 3.9×10^−3^ S cm^−1^ (at 30 °C), respectively,^[64]^ which is often observed with ILs containing an imidazolium cation.^[45]^ Lastly, the IL [N_2222_][DFOB] was reported to have an ionic conductivity of 6.7×10^−3^ S cm^−1^ (at 60 °C)^[64]^ whereas [N_1222_][DFOB] that we synthesised displays an ionic conductivity of 5.9×10^−3^ S cm^−1^ (at 60 °C), reflecting only a small difference upon changing ethyl to a methyl group. Therefore, the conductivity of the new [DFOB]^−^ salts reported here show similar values to those previously reported in the literature, within the same order of magnitude.

The activation energies of ionic transport for [DFOB]^−^‐based salts are shown in Table S2. The activation energies for [P_1222_][DFOB], [P_122i4_][DFOB] and [N_1222_][DFOB] are calculated to be 23, 23, and 24 kJ mol^−1^ respectively, reflecting little impact of cation. On the other hand, Green *et al*. reported a greater activation energy of [P_2444_][DFOB], at 33 kJ mol^−1^,^[65]^ likely as a result of the larger sized phosphonium cation. In the solid state prior to melting, both [P_1222_][DFOB] and [N_1222_][DFOB] display a greater rate of increase in their ionic conductivities with temperature compared to their liquid state. The activation energy for ionic transport in phase I for [C_2_mpyr][DFOB] is 39 kJ mol^−1^, which is the highest of the materials reported here as it exists in the solid state. Furthermore, the activation energy is larger through phase II (E_a_=48 kJ mol^−1^) than in phase I, reflecting the increase in disorder upon heating from phase II to phase I.

### Ionicity

To further understand the properties of the [DFOB]^−^‐based ILs, the Walden Plot was used to qualitatively assess the ionicity by examining the relationship between molar conductivity (Λ) and fluidity (η^−1^). The degree of dissociation between the ions is compared to aqueous KCl (0.01 mol L^−1^), an ideal reference electrolyte that is considered to exhibit complete dissociation.[^66^, ^67^] Recent work by Mariani et al. has also described a plot of ‘reduced ionicity’ whereby 1 M KCl is sometimes suggested for protic ILs,^[68]^ but here we use the format originally proposed by Walden, Watanabe etc.[^66^, ^67^] to make comparisons with previous literature on aprotic salts.

**Figure 6:**
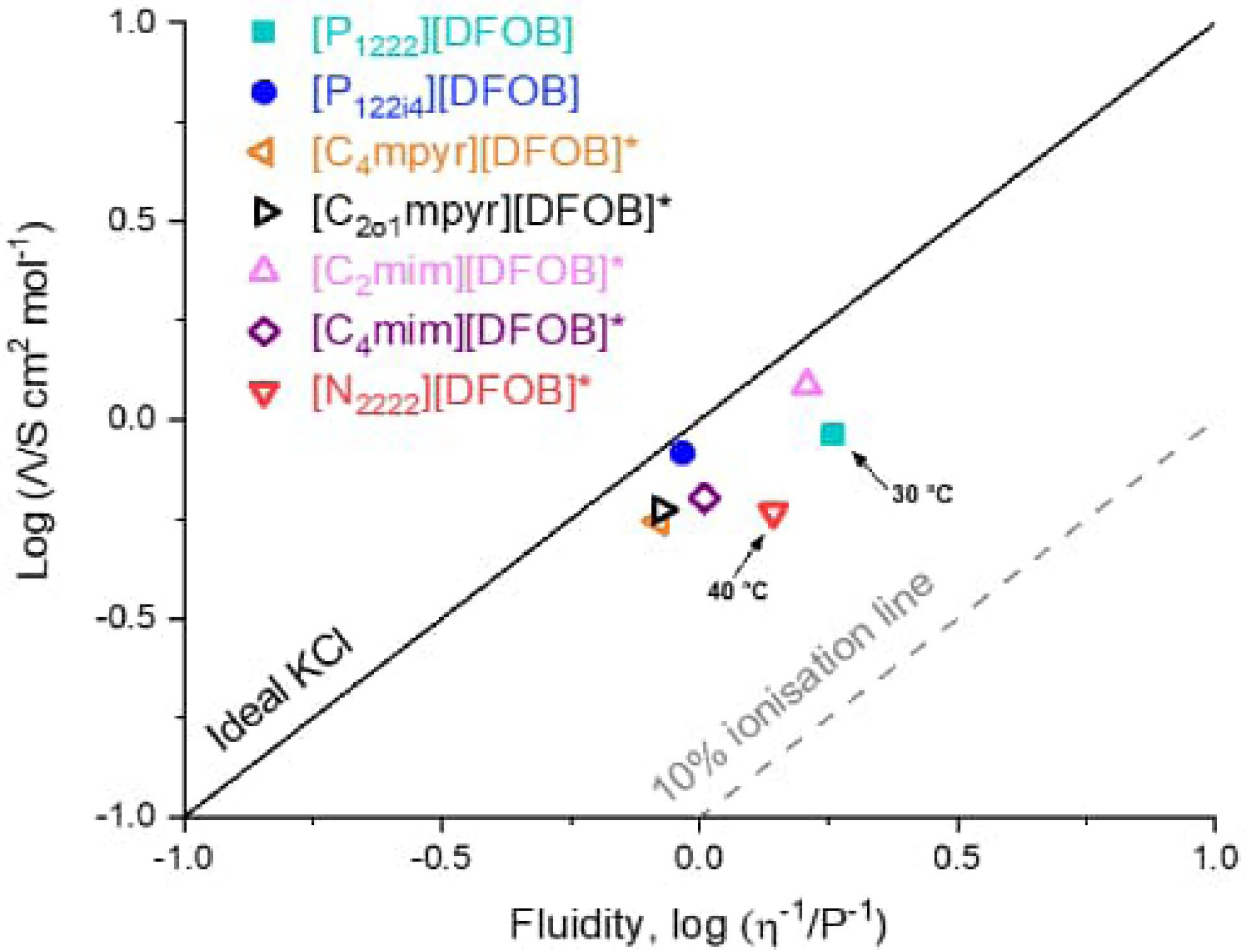
Walden plot of [P_1222_][DFOB] (30 °C) and [P_122i4_][DFOB]. *Comparison with values for [C_4_mpyr][DFOB],^[29]^ [C_2o1_mpyr][DFOB],^[29]^ [C_2_mim][DFOB],^[64]^ [C_4_mim][DFOB]^[64]^ and [N_2222_][DFOB] (40 °C)^[64]^ from the literature are shown. Temperatures are at 25 °C unless otherwise stated.

Figure 6 shows the Walden Plot for ILs [P_1222_][DFOB] and [P_122i4_][DFOB], with comparison to other [DFOB]^−^‐based ILs. The degree of ionicity of ILs can be approximated by the vertical deviation from the ideal KCl line (ΔW), where deviation to below this ideal KCl line represent some degree of ionic association of the IL cations and anions.[^69^, ^70^] Table S3 tabulates the ΔW values with temperature, where ΔW=0 implies complete dissociation whereas ΔW=1 would imply only 10 % dissociation (i. e. substantial ion aggregation).

According to Angell *et al*.,^[71]^ these [DFOB]^−^‐based ILs can be classified as ‘good’ ILs as they lie toward the upper region of the Walden plot near the ideal KCl line. [P_122i4_][DFOB] is observed to exhibit a higher ionicity than [P_1222_][DFOB], which reflects an influence of the isobutyl alkyl chain on improving ion dissociation. The ΔW value of [P_122i4_][DFOB] is lower than [P_1222_][DFOB] (0.06 and 0.30 respectively, at 30 °C). As mentioned earlier, [P_1222_][DFOB] exhibits similar ionic conductivity to [P_122i4_][DFOB], despite its lower viscosity, and the Walden plot indicates that this is due to a larger degree of ion pairing/neutral aggregate formation that do not contribute to the transport of ionic charge.

From the literature data, [C_2_mim][DFOB] displays low ionic association, as commonly observed with imidazolium‐based ILs.^[67]^ Moreover, increasing to a butyl chain with [C_4_mim][DFOB] increases the degree of ion pairing (and viscosity). [C_2o1_mpyr][DFOB] displays a similar ionicity to [C_4_mpyr][DFOB] (within experimental error), which may reflect little effect from the ether functional group present on the alkyl chain. Only small changes in ionicity were previously observed for other alkoxy‐ ILs based on the phosphonium and pyrrolidinium cation when paired with triflate and imide based anions such as [TFSI]^−^ and [FSI]^−^.[^72^, ^73^]

Lastly, the fractional Walden rule can be used to examine the temperature dependence of ion association.[^64^, ^71^] The Walden plot of [DFOB]^−^‐based ILs with increasing temperature is shown in Figure S8. The exponential factor α, a constant between zero and unity, can be used to describe the rate of increase of ion pairing over the given temperature range, where an exponent of α=1 would indicate that the temperature dependence of the molar conductivity is directly proportional to the inverse of the temperature dependent viscosity. An exponent of α=0 would indicate no relationship between the temperature dependent molar conductivity and the viscosity. While α is relatively similar for most of these [DFOB]^−^‐based ILs (0.90 ‐ 0.93), [P_122i4_][DFOB] displays the largest deviation from unity (α=0.847), suggesting that the fluidity increases at a greater rate than the molar conductivity compared to what is expected for aqueous KCl (0.01 mol L^−1^).^[71]^ The observed increase in ΔW values with heating (Table S3) suggests increased ion pairing and/or aggregate formation with temperature for the [DFOB]^−^‐based ILs.

### Electrochemical Stability

**Figure 7:**
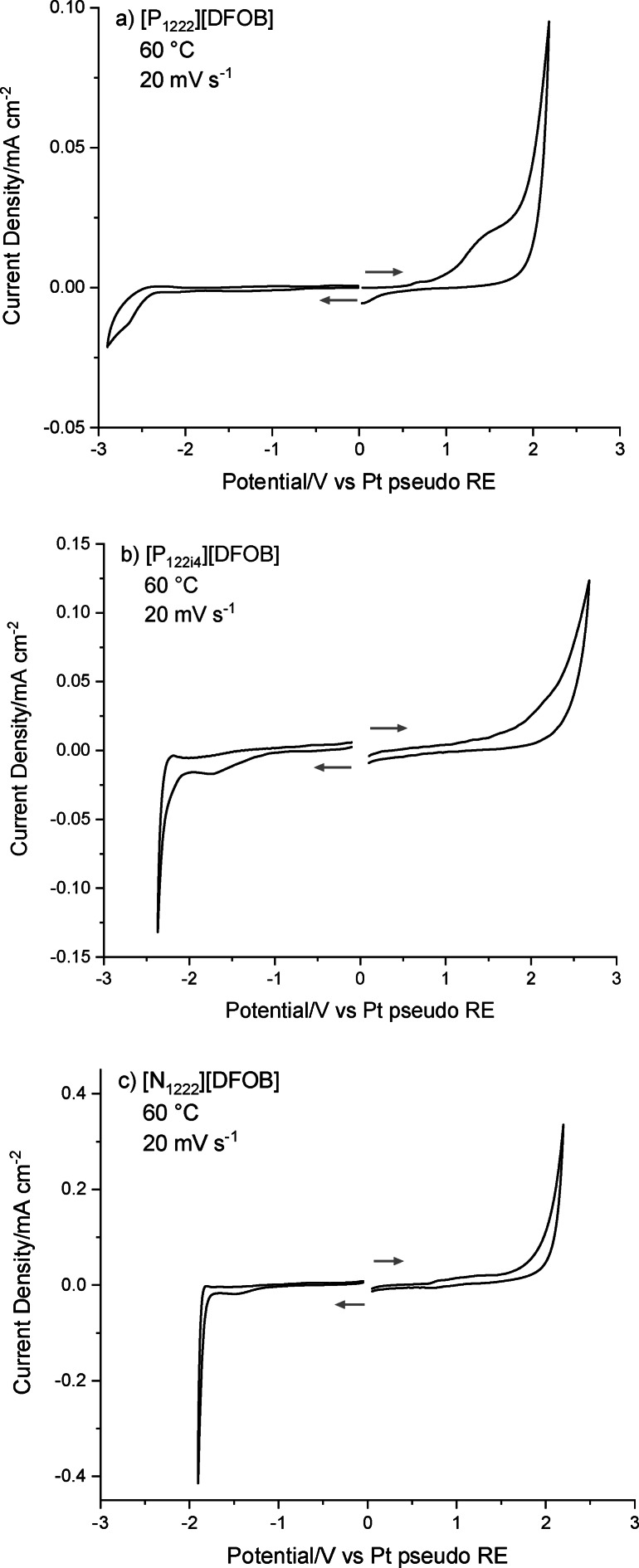
Cyclic voltammetry plots of a) [P_1222_][DFOB], b) [P_122i4_][DFOB] and c) [N_1222_][DFOB] on a Pt working electrode, Pt counter electrode, Pt wire pseudo reference electrode at 60 °C at a scan rate of 20 mV s^−1^. After scanning across positive potentials, all electrodes were cleaned and polished prior to measurement across negative potentials.

The electrochemical stability limits of the new [DFOB]^−^‐based ILs were investigated as an initial assessment of their suitability for use as electrolytes. Experiments were carried out by cyclic voltammetry (CV) using a Pt working electrode, to determine both the oxidative and reductive potential range. A Pt pseudo reference electrode was used for this initial determination of stability, and thus it is the total potential range of stability that is the relevant value rather than the exact cathodic and anodic potential limits. Furthermore, it is proposed that the detrimental impact of halides that would usually lead to lower electrochemical stability^[74]^ is avoided here through use of a tosylate‐based halide‐free route for synthesis of the ILs. An examination of the electrochemical stability of tosylate salts reported an oxidative stability of 3.2 V, based on a glassy carbon working electrode, Pt wire counter electrode and Ag wire pseudo reference electrode.^[46]^ Therefore, it is not considered likely that tosylate salts, if present in trace amounts in the synthesised ILs, would adversely affect the electrochemical stability. The CV plots for three of the [DFOB]^−^‐based ILs are shown in Figure 7a–c.

[P_1222_][DFOB] is observed to display a first oxidation process at >1 V, before the more substantial oxidation process at 2 V. [P_122i4_][DFOB] shows one major oxidation process at >2 V. In terms of reductive stability, both [P_1222_][DFOB] and [P_122i4_][DFOB] display the major reduction peak at a potential of <−2.0 V, vs the Pt pseudo RE. On the other hand, [N_1222_][DFOB] exhibits its major oxidative peak at >1.5 V, and major reduction peak at <−1.7 V.

The electrochemical stability of [C_2_mpyr][DFOB] could not be determined due to its low conductivity at 60 °C (2×10^−6^ S cm^−1^), and poor contact with the electrodes as it exists as a powdery solid. However, it is important to note that the addition of Li/Na salt to [C_2_mpyr][DFOB], to form electrolytes for Li or Na batteries, would be expected to increase the conductivity by orders of magnitude,[^2^, ^75^] and may also increase the electrochemical stability.

In prior literature, Allen and co‐workers reported the electrochemical stability of [C_4_mpyr][DFOB] to be 5.0 V (vs. Li/Li^+^). However, the authors observed unusual partially reversible redox behaviour on the cathodic scan for [C_4_mpyr][DFOB]. In contrast, such behaviour was never observed in our [DFOB]^−^‐based ILs. In summary, preliminary electrochemical studies of our halide‐free [DFOB]^−^‐based ILs show sufficiently promising electrochemical stability to encourage future investigation into their use for Li or Na electrochemistry.

## Conclusions

The synthesis of new ILs and OIPCs based on the [DFOB]^−^ anion was successfully achieved with high purity using a halide‐free tosylate route. The new synthetic method is operationally straight forward and amenable to scale up. Given the impact of trace halides on ILs and OIPCs performance, this tosylate‐based method should be applied to the synthesis of other small ILs and OIPCs where traditional methods are not suitable. The series of new electrolytes developed here are designed to exhibit the advantages of the [DFOB]^−^ anion, such as good SEI forming ability, Al passivation and higher salt solubilities, along with the beneficial properties of ILs and OIPCs that are non‐flammable and non‐volatile, combined into a neat liquid or solid material.

The new ILs [P_1222_][DFOB] and [P_122i4_][DFOB], and OIPCs [N_1222_][DFOB] and [C_2_mpyr][DFOB], were characterised in terms of their thermal behaviour and transport properties. All of the [DFOB]^−^‐based salts demonstrated good thermal stability and exhibited at least one large solid‐solid phase transition prior to melting. Interestingly, [C_2_mpyr][DFOB] was found to be a higher melting OIPC compared to [N_1222_][DFOB] due to the cyclic pyrrolidinium cationic structure vs the non‐cyclic ammonium cation. The [DFOB]^−^‐based ILs displayed relatively high ionic conductivity, where those with the phosphonium cation showed higher values. Furthermore, these ILs demonstrated exhibited good electrochemical stability windows. Hence, further investigations into the use of the [DFOB]^−^ anion to form IL and OIPC‐based electrolytes, with added lithium or sodium salts, may overcome the shortfalls of existing electrolytes towards designing robust electrolytes for next‐generation batteries.

## Experimental Section

### Materials

Triethylmethylammonium chloride ([N_1222_][Cl] (>97 %, Sigma Aldrich), lithium difluoro(oxalato)borate ([Li][DFOB]) (Sigma Aldrich), N,N‐ethyl‐methylpyrrolidinium 4‐methylbenzenesulfonate ([C_2_mpyr][Ts]) (>99 %, Boron Molecular), *N*‐methylpyrrolidine (>99.5 %, Sigma Aldrich), bromoethane (>98 %, Sigma Aldrich), triethylmethylphosphonium tosylate ([P_1222_][Ts], >98 % Cytec Industries), isobutyldiethylmethylphosphonium ([P_122i4_][Ts], >98 %, Cytec Industries), methyl *p*‐toluenesulfonate (>99 %, Boron Molecular), triethylamine (Boron Molecular) were used as received. All OIPCs and ILs were dried for at least 48 hours at room temperature under vacuum (*ca*. 0.1 mbar) in order to ensure that water had been completely removed, with subsequent handling and sample preparation in an Argon glove box.

### Characterisation

^1^H (400 MHz), ^19^F (375 MHz), ^11^B (128 MHz) and ^31^P (162 MHz) NMR spectra were measured on a Bruker Avance III instrument using deuterated dimethylsulfoxide ((CD_3_)_2_SO) as a solvent. For all OIPCs and ILs, no impurity peaks were observed (e. g. CH_3_CN, BF(OH)_3_, BF_2_(OH)_2_, BF_3_OH etc.). Water content could not be determined with [DFOB]^−^‐based salts due to side reactions with the oxalato functional group on the [DFOB]^−^ anion and the Karl‐Fischer Titration solution. Mass spectrometry data was obtained using an Agilent 1200 series HPLC system. Elemental analysis (C, H, N) was carried out at Monash Analytical Platform (School of Chemistry). Lithium content was quantified using inductively coupled plasma‐mass spectrometry (ICP‐MS, NexION 350X, PerkinElmer, USA). Lithium calibration standards were prepared between a range of 0.1–500 ppb containing 2 % suprapur nitric acid. The ICP‐MS was operated under kinetic energy discrimination mode with 50 ms dwell times, 20 sweeps, one reading and three replicates. The plasma source conditions were run using: nebuliser gas flow 1.02 L min^−1^, auxiliary gas flow 1.2 L min^−1^, plasma gas flow 15 L min^−1^, and ICP RF power 1500 W. Synhistix (PerkinElmer) software was utilised to analyse data.

The melting point of the solid samples were initially determined using a Gallenkamp melting point apparatus prior to Differential Scanning Calorimetry (DSC) measurements. A Mettler Toledo DSC STARe instrument was used to conduct such experiments at a scan rate of 10 °C min^−1^_,_ unless specified otherwise, under N_2_ flow. Thermogravimetric Analysis (TGA) was measured using a Mettler Toledo TGA/DSC 1 STARe System from 25–550 °C under N_2_ flow of 30 mL min^−1^ at a heating rate of 10 °C min^−1^. Samples for DSC and TGA were prepared in aluminium pans (Mettler Toledo) in a glovebox under argon (Ar) atmosphere.

Electrochemical impedance spectroscopy was used to determine the ionic conductivity of samples using a Biologic MTZ35 Solartron Modulab. The conductivity of liquids or soft solids was measured using a custom‐designed ‘dip cell’ with two platinum electrodes. The cell constant was determined with 10 mM KCl_(aq)_ at 30 °C. Solid samples were pressed into pellets (5 ton), under Ar, and sandwiched between two stainless steel electrode plates housed in a custom‐designed barrel cell. The total resistance determined by fitting the semi‐circle of the Nyquist plot, and known dimensions of the pellet for solid samples, allowed the determination of the ionic conductivity values. The density and viscosity of the low melting samples were measured using an Anton Paar: DMA5000, LOVIS/2000ME (with data shown in the supporting information). The [N_1222_][DFOB] could not be analysed because it has a melting point of 50 °C. The molar conductivity (Λ, S cm^2^ mol^−1^) was calculated through the parameters of ionic conductivity (σ , S cm^−1^), density (ρ , g cm^−3^) and the molecular weight of the salt (MW, g mol^−1^) as per the equation:

$$\Lambda =\frac{\sigma }{\rho }\;\mathrm{MW}$$

Cyclic voltammetry (CV) experiments were carried out using a three‐electrode cell: a platinum disk as the working electrode, a coiled platinum wire for the counter electrode, and a platinum wire as the pseudo‐reference electrode. All electrodes were cleaned with alumina and water, sonicated and dried after each scan in the positive or negative direction. CV analysis was carried out on a Biologic SP‐200 potentiostat inside a glovebox under an Ar environment.

### Synthesis

#### N‐ethyl‐N‐methylpyrrolidinium 4‐methylbenzenesulfonate ([C_2_mpyr][Ts])

Ethyl benzenesulfonate (10.00 g, 58.1 mmol) was dissolved in acetone and heated to reflux. *N*‐methylpyrrolidine (5.10 g, 59.9 mmol) was added dropwise over 30 minutes and the solution refluxed overnight. After cooling to room temperature, the mixture was further cooled in an ice bath and the solid isolated by filtration in vacuo. The white solid was washed with cold acetone (50 mL) three times and dried in vacuo for 1 hour. The solid was further dried in vacuo at 70 °C overnight to afford a white solid (12.0 g, 72 %)

^1^H NMR (400 MHz, CDCl_3_): 1.21–1.29 (t, *J*_*HH*=_7.4 Hz, CH_2_ C *H*_3_, 3H), 2.04–2.12 (q, CH_2_ CH_2_, 4H), 2.30 (s, C_6_H_4_ CH_3_, 3H), 3.00 (s, NCH_3_, 3H), 3.41–3.48 (q, NCH_2_, 2H), 3.45–3.55 (m, N(CH_2_CH_2_)_2_, 4H); 7.08–7.12 (d, *J_HH_* =7.9 Hz, CH_3_ C_6_ H_4_, 2H), 7.67–7.71 (d, *J_HH_* =8.1 Hz, C_6_ H_4_SO_3_ 2H); ES^+^ *m*/*z* 116 (C_7_H_18_N)^+^, 87.1 (C_5_H_13_N)^+^, 72.0 (C_4_H_10_N)^+^. ES^−^ *m*/*z* 171.0 (C_7_H_7_SO_3_)^−^.

#### N‐ethyl‐N‐methylpyrrolidinium bromide ([C_2_mpyr][Br])

The synthesis of [C_2_mpyr][Br] was based on a literature method.^[76]^ *N*‐methylpyrrolidine (4.42 g, 51.9 mmol) was dissolved in dry ethyl acetate (15 mL) and cooled in an ice bath, followed by the dropwise addition of a solution of bromoethane (5.95 g, 54.6 mmol) in dry ethyl acetate (20 mL). After the formation of a cloudy solution (1 hour), the mixture was heated to 30 °C and stirred under Ar for 24 hours. After filtration of the solid under Ar, the white solid was rinsed three times with cold, dry ethyl acetate followed by drying in vacuo to afford a white solid (7.43 g, 74 %). ^1^H NMR (400 MHz, DMSO): 1.24–1.29 (tt, *J*_*HH*=_7.3 Hz, CH_2_ C *H*_3_, 3H), 2.04–2.10 (m, N(CH_2_ CH_2_)_2_, 4H), 2.97 (s, NCH_3_, 3H); 3.36–3.41 (q, *J_HH_* =7.3 Hz, NCH_2_CH_3_, 2H), 3.40–3.50 (m, N(CH_2_CH_2_)_2_, 4H). ES^+^ *m*/*z* 114.0 (C_7_H_16_N)^+^, ES^−^ *m*/*z* 78.7, 80.7 (Br)^−^

#### Triethylmethylammonium 4‐methylbenzenesulfonate ([N_1222_][Ts])

A solution of triethylamine (7.53 g, 74 mmol) in acetone (30 mL) was added dropwise to a solution of methyl 4‐methylbenzenesulfonate (13.2 g, 71 mmol) in acetone (30 mL) at 40 °C under Ar. After complete addition, the mixture was refluxed overnight at 60 °C under Ar. After cooling to room temperature, acetone was mostly removed under reduced pressure. Methyl tert‐butyl ether (50 mL) was added to precipitate out a white solid followed by stirring over a few hours. The solid was isolated by filtration in vacuo and rinsed with cold methyl tert‐butyl ether to afford a white solid (19.70 g, 97 %).

^1^H NMR (400 MHz, DMSO): 1.15–1.22 (t, *J_HH_* =7.2 Hz, (C*H*_3_)_3_, 9H), 2.29 (s, C_6_H_4_ CH_3_, 3H), 2.87 (q, NCH_3_, 3H), 3.19–3.30 (q, *J_HH_* =7.3 Hz, N(CH_2_)_3_, 6H), 7.09–7.13 (d, *J_HH_* =7.9 Hz, CH_3_ C_6_ H_4_, 2H), 7.45–7.49 (d, *J_HH_* =8.1 Hz, C_6_ H_4_SO_3_ 2H); ES^+^ *m*/*z* 116 (C_7_H_18_N)^+^, 87.1 (C_5_H_13_N)^+^, 72.0 (C_4_H_10_N)^+^. ES^−^ *m*/*z* 171.0 (C_7_H_7_SO_3_)^−^.

#### Triethylmethylammonium difluoro(oxalato)borate ([N_1222_][DFOB])

LiDFOB (4.48 g, 31.2 mmol) was dissolved in dry acetonitrile (30 mL) and added dropwise to a solution of [N_1222_][Ts] (8.96 g, 31.2 mmol) in dry acetonitrile (30 mL) cooled in an ice‐bath and left to stir overnight under Ar. The white precipitate, which formed immediately upon LiDFOB addition, was filtered out under Ar after which the acetonitrile was removed in vacuo. After the addition of DCM (50 mL) to the dried filtrate, the solution was left to cool in the freezer followed by the filtration through a syringe filter (0.22 μm, PTFE) to remove any remaining particles. Activated charcoal was added and the mixture was left to stir overnight. After the charcoal was filtered out, the solution was washed with water (5 mL) once, after which DCM was removed in vacuo to afford a white opaque solid (3.12 g, 40 %).

^1^H NMR (400 MHz, DMSO): 1.17–1.21 (tt, *J_HH_* =7.4 Hz, (C*H*_3_)_3_, 9H), 2.87 (s, NCH_3_, 3H), 3.22–3.27 (q, N(CH_2_)_3_, 6H); ^19^F NMR (376 MHz, DMSO): −150.8 (BF_2_); ^11^B NMR (128 MHz, DMSO): 2.92 (B); ES^+^ *m*/*z* 116.1 (C_7_H_18_N)^+^, 87.0 (C_5_H_13_N)^+^, 72.0 (C_4_H_10_N)^+^. ES^−^ *m*/*z* 137 (BF_2_C_2_O_4_)^−^, 92.9 (BF_2_CO_2_)^−^, 86.8 (BF_4_)^−^, 64.9 (BOF_2_)^−^. Anal. calculated for C_9_H_18_NBF_2_O_4_: C, 42.72; H, 7.17; N, 5.54. Found: C, 42.48; H, 7.37; N, 5.56. Lithium content (ICP): 196 ppm. Visual melting point: 50 °C.

#### N‐ethyl‐N‐methylpyrrolidinium difluoro(oxalato)borate ([C_2_mpyr][DFOB])

LiDFOB (4.03 g, 28.0 mmol) was dissolved in dry acetonitrile (35 mL) and added dropwise to a solution of [C_2_mpyr][Ts] (8.00 g, 28.0 mmol) in dry acetonitrile (35 mL) cooled in an ice‐bath and left to stir overnight under Ar. The white precipitate, that formed immediately, was filtered out under Ar where acetonitrile was removed in vacuo. After the addition of DCM (50 mL) to the dried filtrate, the solution was left to cool in the freezer followed by the filtration through a syringe filter (0.22 μm, PTFE) to remove any remaining particles. Activated charcoal was added and the solution was left to stir overnight. After the charcoal was filtered out, the solution was washed with water (5 mL) once, after which DCM was removed in vacuo. The solid was dissolved in dry acetonitrile (2 mL) followed by the addition of diethyl ether (10 mL) upon which the bottom layer was separated and dried in vacuo to afford a white solid (2 g, 28 %).

^1^H NMR (400 MHz, DMSO): 1.24–1.29 (tt, *J*_*HH*=_7.4 Hz, (C*H*_3_)_3_, 3H), 2.07 (m, N(CH_2_ CH_2_)_2_, 4H), 2.95 (s, NCH_3_, 3H), 3.33–3.39 (q, NCH_2_, 2H), 3.41–3.48 (m, N(CH_2_CH_2_)_2_, 4H); ^19^F NMR (376 MHz, DMSO): −150.8 (BF_2_); ^11^B NMR (128 MHz, DMSO): 2.91 (B). ES^+^ *m*/*z* 114.1 (C_7_H_15_N)^+^, ES^−^ *m*/*z* 137 (BF_2_C_2_O_4_)^−^, 114.9 (BC_2_O_5_)^−^, 92.9 (BF_2_CO_2_)^−^, 86.8 (BF_4_)^−^, 64.9 (BOF_2_)^−^. Anal. calculated for C_9_H_16_NBF_2_O_4_: C, 43.06; H, 6.43; N, 5.58;. Found: C, 43.07; H, 6.48; N, 5.63. Lithium content (ICP): 23 ppm. Visual melting point 203–204 °C.

#### Diethyl(methyl)(isobutyl)phosphonium difluoro(oxalato)borate ([P_122i4_][DFOB])

LiDFOB (3.19 g, 22.2 mmol) was dissolved in dry acetonitrile (35 mL) and added dropwise to a solution of [P_122i4_][Ts] (7.04 g, 21.2 mmol) in dry acetonitrile (35 mL) cooled in an ice‐bath and left to stir overnight under Ar. The white precipitate, that formed immediately, was filtered out under Ar where acetonitrile was removed in vacuo. After the addition of DCM (50 mL) to the dried filtrate, the solution was left to cool in the freezer followed by the filtration through a syringe filter (0.22 μm, PTFE) to remove any remaining particles. Activated charcoal was added and the solution was left to stir overnight. After charcoal was filtered out, the solution was washed with water (5 mL) once, after which DCM was removed in vacuo. The solid was dissolved in dry acetonitrile (2 mL) followed by the addition of diethyl ether (10 mL) upon which the bottom layer was separated and dried in vacuo to afford a white solid (2.48 g, 39 %).

^1^H NMR (400 MHz, DMSO): 1.02–1.03 (d, *J*_*HH*=_6.6 Hz, *J_PH_* =0.8 Hz, CH(C *H*_3_ )_2_, 6H), 1.09–1.17 (dt, *J_HH_* =7.6 Hz, *J_PH_* =18.6 Hz, P(CH_2_ CH_3_)_2_, 6H), 1.79–1.83 (d, *J_PH_* =13.9 Hz, PCH_3_, 3H), 1.95–2.07 (m, CH, H), 2.12–2.24 (m, P(CH_2_)_3_, 6H); ^19^F NMR (376 MHz, DMSO): −150.8 (BF_2_); ^11^B NMR (128 MHz, DMSO): 2.92 (B); ^31^P NMR (162 MHz, DMSO): 34.9 (P); ES^+^ *m*/*z* 162.2 (C_9_H_22_P)^+^, 147.1 (C_5_H_13_P)^+^, 105.0 (C_8_H_19_P)^+^. ES^−^ *m*/*z* 137 (BF_2_C_2_O_4_)^−^, 114.9 (BC_2_O_5_)^−^, 92.9 (BF_2_CO_2_)^−^, 86.8 (BF_4_)^−^, 64.9 (BOF_2_)^−^, 186.9 (BC_4_O_8_)^−^. Lithium content (ICP): 88 ppm.

#### Triethyl(methyl)phosphonium difluoro(oxalato)borate ([P_1222_DFOB])

LiDFOB (5.02 g, 34.9 mmol) was dissolved in dry acetonitrile (35 mL) and added dropwise to a solution of [P_1222_][Ts] (10.41 g, 34.2 mmol) in dry acetonitrile (35 mL) cooled in an ice‐bath and left to stir overnight under Ar. The white precipitate, that formed immediately, was filtered under Ar where acetonitrile was removed in vacuo. After the addition of DCM (50 mL) to the dried filtrate, the solution was left to cool in the freezer followed by the filtration through a syringe filter (0.22 μm, PTFE) to remove any remaining particles. Activated charcoal was added and the solution was left to stir overnight. After filtration, the solution was washed with water (5 mL) once, after which DCM was removed in vacuo. The solid was dissolved in dry acetonitrile (2 mL) followed by the addition of diethyl ether (10 mL) upon which the bottom layer was separated and dried in vacuo to afford a white solid (4.17 g, 45 %).

^1^H NMR (400 MHz, DMSO): 1.08–1.16 (dt, *J*_*HH*=_7.7 Hz, *J_PH_* =18.6 Hz, (C*H*_3_)_3_, 9H), 1.74–1.78 (d, *J_PH_* =14.0 Hz, PCH_3_, 3H), 2.13–2.22 (m, P(CH_2_)_3_, 6H); ^19^F NMR (376 MHz, DMSO): −150.8 (BF_2_); ^11^B NMR (128 MHz, DMSO): 2.90 (B); ^31^P NMR (162 MHz, DMSO): 37.9 (P). ES^+^ *m*/*z* 134.2 (C_7_H_18_P)^+^, 90.0 (C_4_H_10_P)^+^. ES^−^ *m*/*z* 137.0 (BF_2_C_2_O_4_)^‐^, 92.9 (BF_2_CO_2_)^−^, 64.9 (BOF_2_)^−^. Lithium content (ICP): 273 ppm

## Conflict of interest

The authors declare no conflict of interest.

1

## Supporting information

As a service to our authors and readers, this journal provides supporting information supplied by the authors. Such materials are peer reviewed and may be re‐organized for online delivery, but are not copy‐edited or typeset. Technical support issues arising from supporting information (other than missing files) should be addressed to the authors.

Supporting Information

## Data Availability

The data that support the findings of this study are available from the corresponding author upon reasonable request.
