# Supplementary material for: Halide‐Free Synthesis of New Difluoro(oxalato)borate [DFOB]−‐Based Ionic Liquids and Organic Ionic Plastic Crystals
Source: Chemphyschem. 2022 May 17;23(13):e202200115. doi: 10.1002/cphc.202200115 (PMC9401595; doi:10.1002/cphc.202200115)
Supplement: Supplementary file 1 — Supporting Information [file CPHC-23-0-s001.pdf]

ChemPhysChem

Supporting Information

Halide-Free Synthesis of New Difluoro(oxalato)borate $[DFOB]^-$-Based Ionic Liquids and Organic Ionic Plastic Crystals

Colin S. M. Kang, Oliver E. Hutt, and Jennifer M. Pringle*

Supporting Information

General Synthesis:

General details that affect the synthesis and characterisation should be noted with regard to [DFOB]$^-$ -based salts. First, for the determination of halide-based impurities, it was found that the [DFOB]$^-$ anion interacts with 0.1 M $AgNO_3$ solution to produce a white precipitate. This will lead to a false positive when trying to determine the presence of halide content. In addition, the [DFOB]$^-$ anion directly interacted with Br^- ISE (Ion Selective Electrode).

The dropwise addition of Li[DFOB] (in acetonitrile) is recommended since fast addition can result in a more intensely coloured product. In terms of impurities, trace Li^+ content can be removed via charcoal, tosylate-based impurities can be removed via water washing (low volume, due to the hydrophilic products), and the occasional appearance of ^{1}H NMR peaks at 4.0–5.2 ppm can be removed via acetonitrile/diethyl ether extraction.

Table S1: Table of ^{19}F and ^{11}B peak integral values indicating the ratio of each species present.

	**^{19}F**			**^{11}B**		
	[DFOB]$^-$	$[BF_4]^-$		[DFOB]$^-$	[BOB]$^-$	$[BF_4]^-$
$[P_{1222}]$[DFOB]	1.0000	0.0036		1.0000	0.0021	0.0008
$[P_{122i4}]$[DFOB]	1.0000	0.0060		1.0000	0.0223	0.0029
$[N_{1222}]$[DFOB]	1.0000	0.0032		1.0000	0.0011	0.0014
$[C_2mpyr]$[DFOB]	1.0000	0.0015		1.0000	0.0007	0.0008

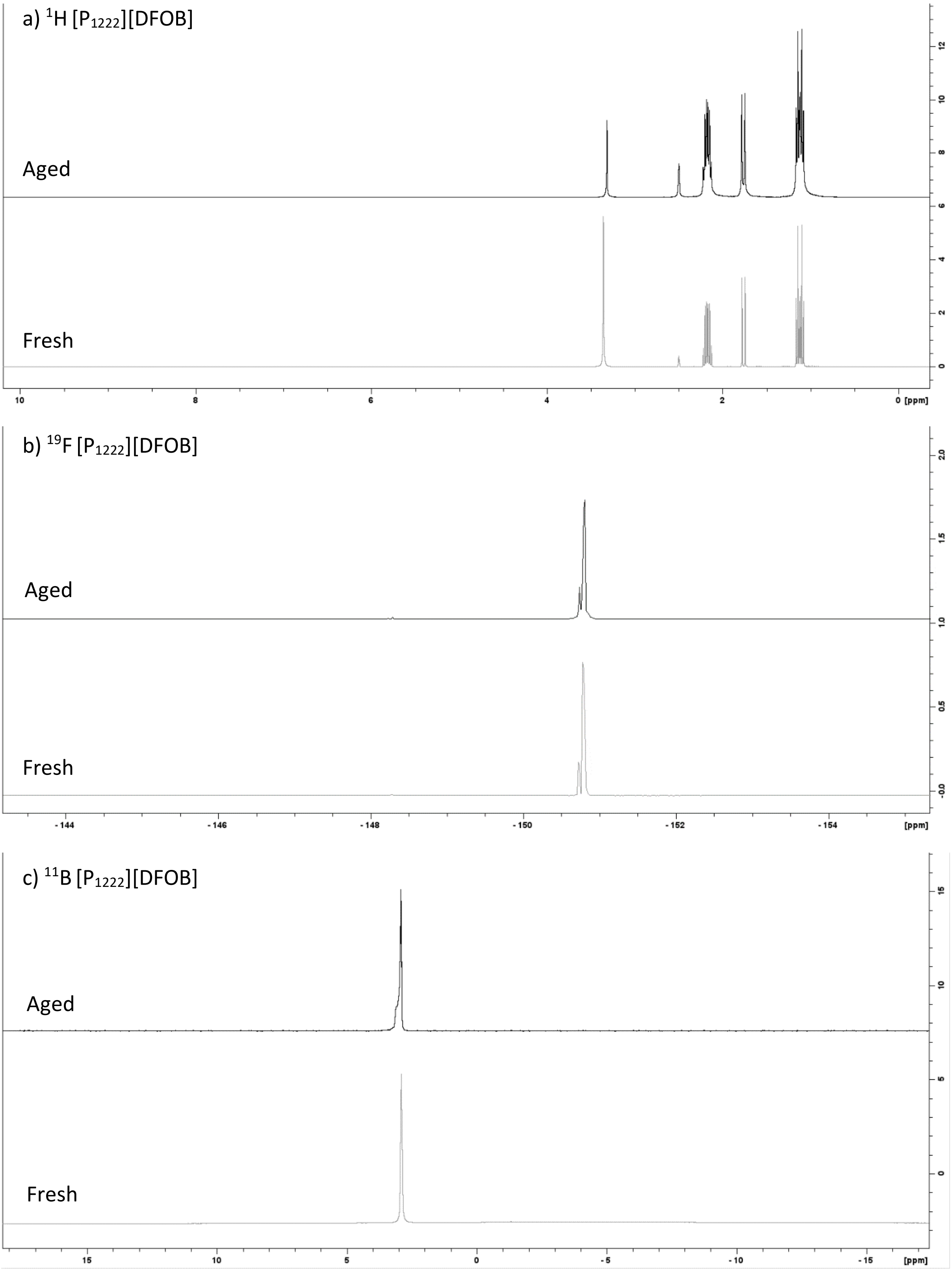

Figure S1: a) ^{1}H NMR, b) ^{19}F NMR, and c) ^{11}B NMR spectra of $[P_{1222}][DFOB]$ of 'aged' samples (above) versus 'freshly' prepared samples (below)

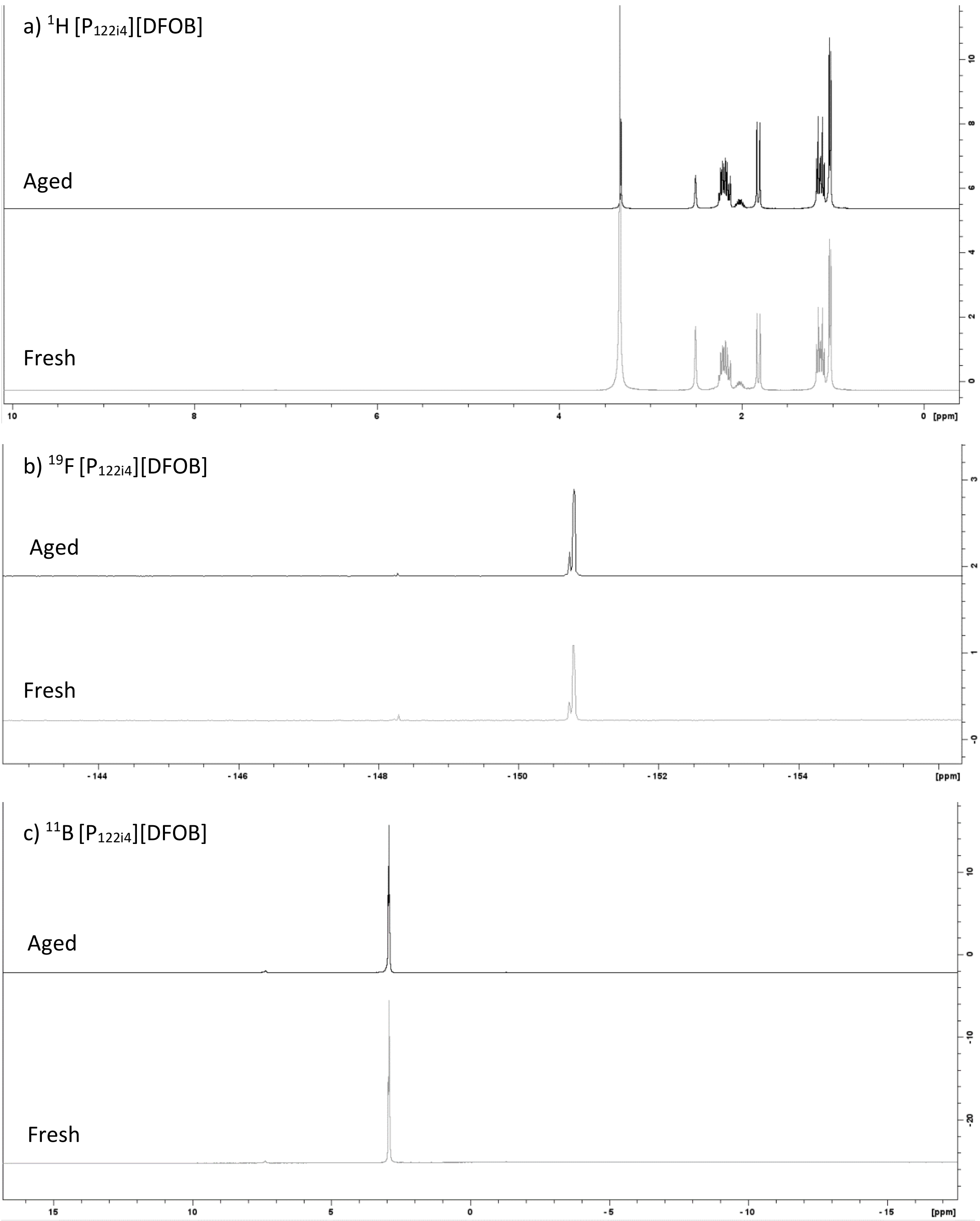

Figure S2: a) ^{1}H NMR, b) ^{19}F NMR, and c) ^{11}B NMR spectra of [P_{122i4}][DFOB] of 'aged' samples (above) versus 'freshly' prepared samples (below)

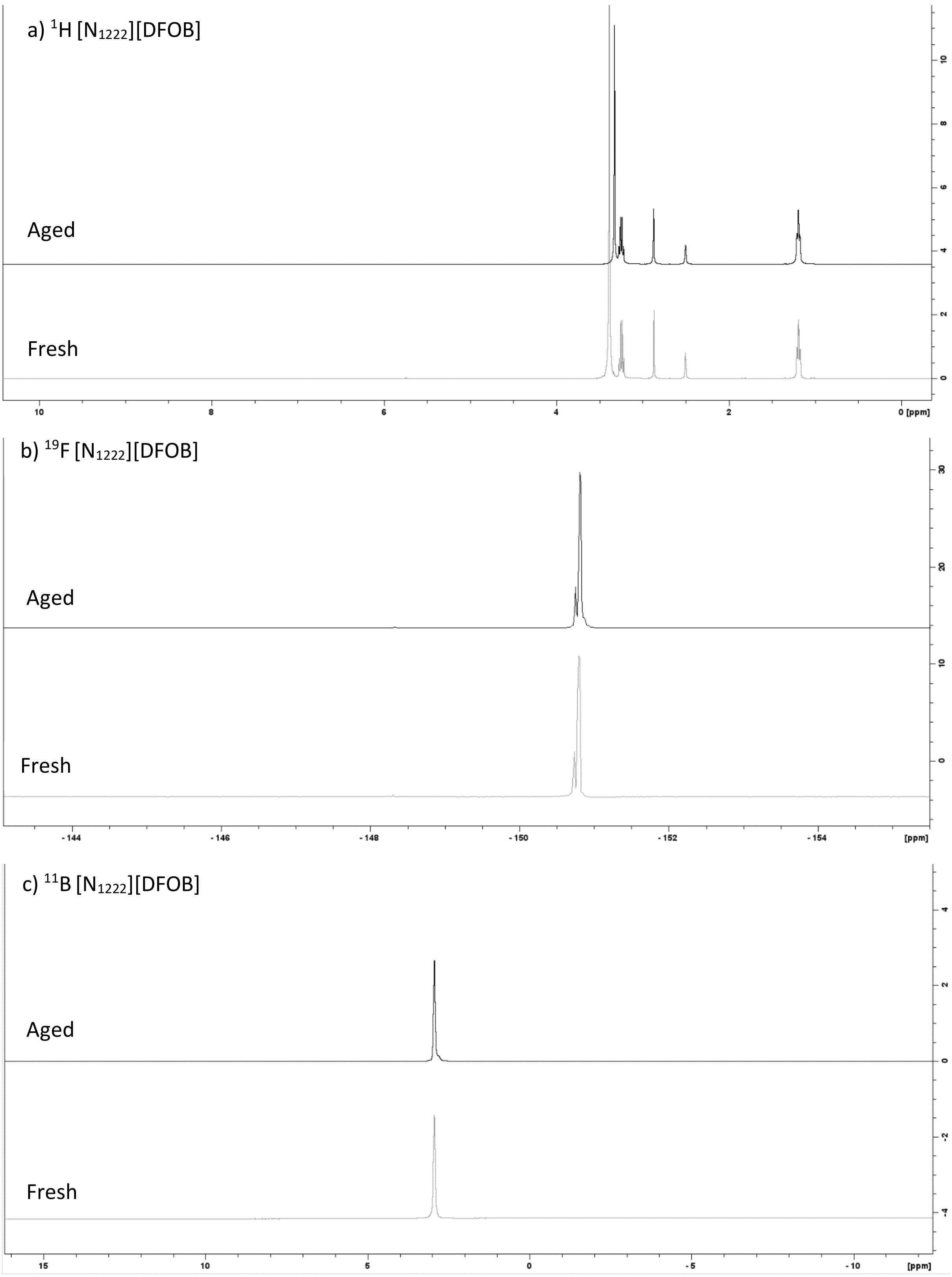

Figure S3: a) ^{1}H NMR, b) ^{19}F NMR, and c) ^{11}B NMR spectra of $[N_{1222}][DFOB]$ of ‘aged’ samples (above) versus ‘freshly’ prepared samples (below)

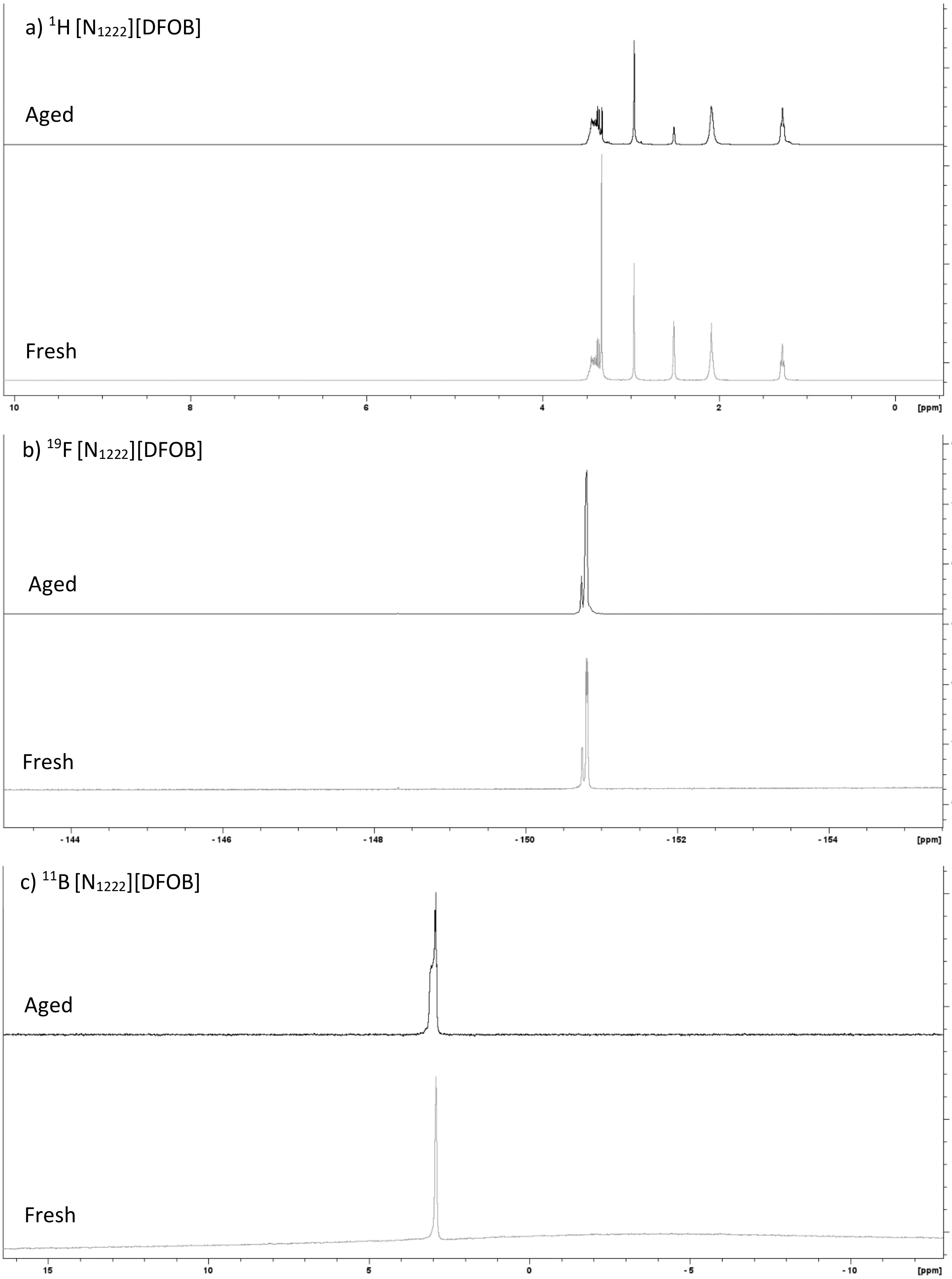

Figure S4: a) ^{1}H NMR, b) ^{19}F NMR, and c) ^{11}B NMR spectra of [C_2mpyr][DFOB] of 'aged' samples (above) versus 'freshly' prepared samples (below)

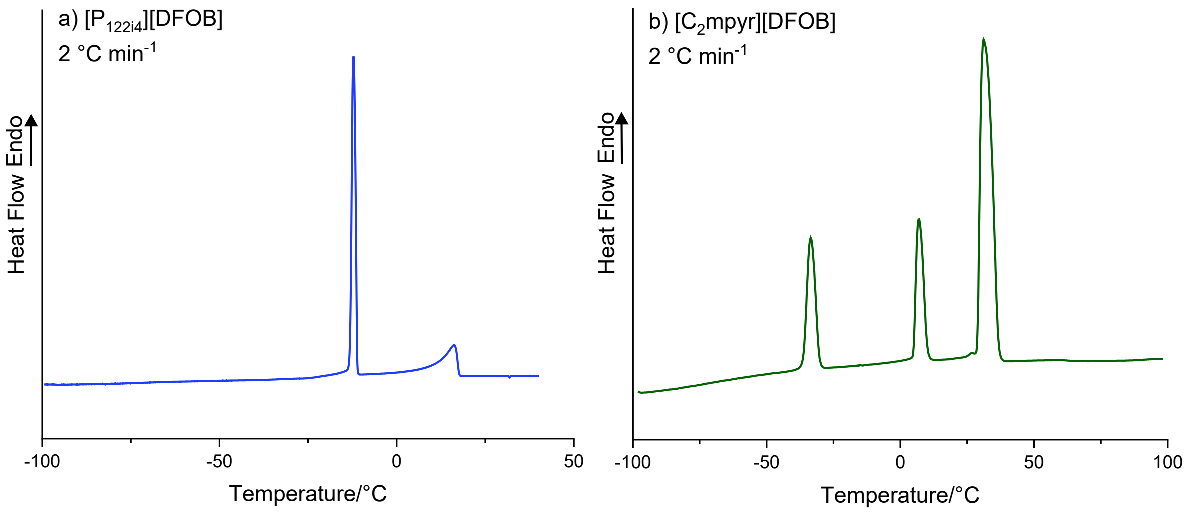

Figure S5: Differential Scanning Calorimetry traces for a) [P_{122i4}][DFOB] and b) [C_2mpyr][DFOB] at a scan rate of 2 °C min^{-1}

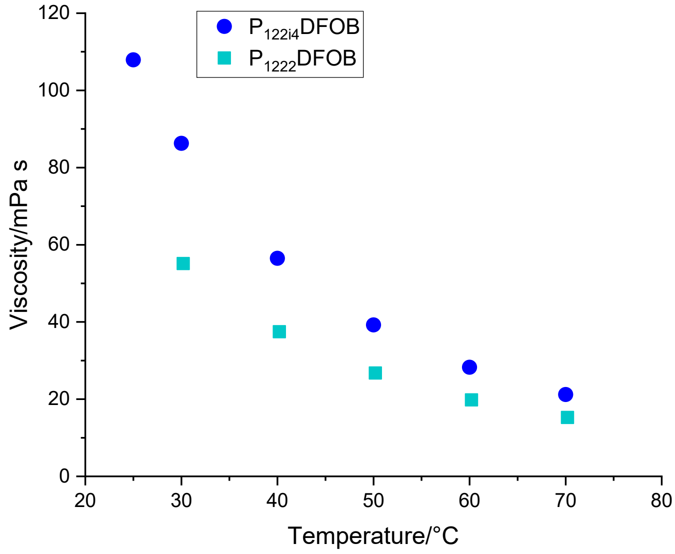

Figure S6: Plot of viscosity vs temperature for [P_{1222}][DFOB] and [P_{122i4}][DFOB]

Table S2: Ionic conductivities at 50 °C and activation energies for [DFOB]$^-$-based salts

	Ionic Conductivity /S cm^{-1} ± 5%	Activation Energy /kJ mol^{-1} ± 5%
**[P_{1222}][DFOB]**	7.1 x 10^{-3}	24
**[P_{122i4}][DFOB]**	6.9 x 10^{-3}	23
**[N_{1222}][DFOB]**	2.5 x 10^{-3}	23
**[C_2mpyr][DFOB]**	1.0 x 10^{-6}	39 (phase I)<br>48 (phase II)

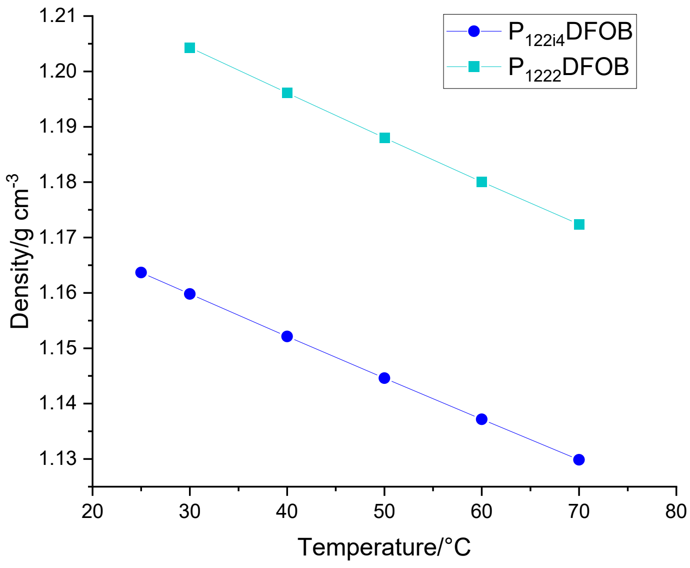

Figure S7: Plot of density vs temperature for [P_{1222}][DFOB] and [P_{122i4}][DFOB]

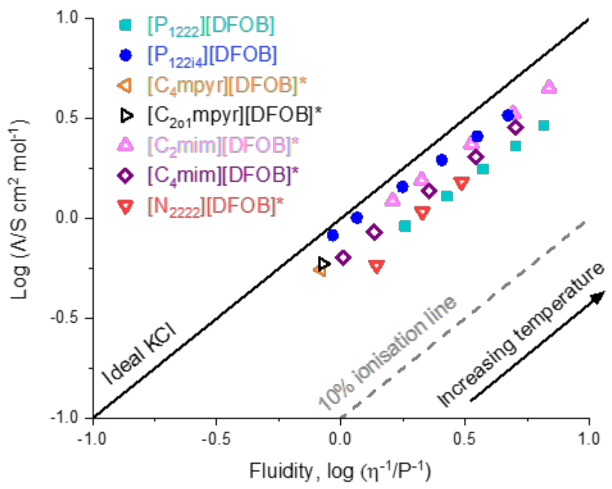

Figure S8: Temperature dependent Walden plot for [P_{1222}][DFOB] (30 - 70 °C) and [P_{122i4}][DFOB] (25, 30 - 70 °C). *Comparison with data for [C_4mpyr][DFOB] (25 °C),[1] [C_{2o1}mpyr][DFOB] (25 °C),[1] [C_2mim][DFOB] (25, 30 - 70 °C),[2] [C_4mim][DFOB] (25, 30 - 70 °C),[2] and [N_{2222}][DFOB] (40 - 70 °C)[2] are shown.

Table S3: ΔW change with temperature and the slope of fractional Walden plot

	Temperature /°C	Deviation from ideal KCl line ΔW ± 0.05	Exponent factor/α
**[P_{1222}][DFOB]**	30	0.30	0.902 ± 0.001
	40	0.31	
	50	0.33	
	60	0.34	
	70	0.35	
**[P_{122i4}][DFOB]**	25	0.05	0.847 ± 0.003
	30	0.06	
	40	0.09	
	50	0.11	
	60	0.14	
	70	0.16	
**[C_4mpyr][DFOB]**[1]	25	0.18	-
**[C_{2o1}mpyr][DFOB]**[1]	25	0.15	-
**[C_2mim][DFOB]**[2]	25	0.12	0.898 ± 0.002
	30	0.13	
	40	0.15	
	50	0.17	
	60	0.19	
**[C_4mim][DFOB]**[2]	25	0.19	0.928 ± 0.001
	30	0.20	
	40	0.22	
	50	0.23	
	60	0.25	
**[N_{2222}][DFOB]**[2]	40	0.28	0.933 ± 0.002
	50	0.29	
	60	0.30	

References

[1] M. Amereller, T. Schedlbauer, D. Moosbauer, C. Schreiner, C. Stock, F. Wudy, S. Zugmann, H. Hammer, A. Maurer, R. M. Gschwind, H. D. Wiemhöfer, M. Winter, H. J. Gores, *Prog. Solid State Chem.* **2014**, *42*, 39–56.

[2] C. Schreiner, S. Zugmann, R. Hartl, H. J. Gores, *J. Chem. Eng. Data* **2010**, *55*, 4372–4377.
